# Digital Holographic Microscopy in Veterinary Medicine—A Feasibility Study to Analyze Label-Free Leukocytes in Blood and Milk of Dairy Cows

**DOI:** 10.3390/ani14213156

**Published:** 2024-11-03

**Authors:** Sabine Farschtschi, Manuel Lengl, Stefan Röhrl, Christian Klenk, Oliver Hayden, Klaus Diepold, Michael W. Pfaffl

**Affiliations:** 1 Division of Animal Physiology and Immunology, TUM School of Life Sciences, Technical University of Munich, 85354 Freising, Germany; michael.pfaffl@tum.de; 2 Chair of Data Processing, TUM School of Computation, Information and Technology, Technical University of Munich, 80333 Munich, Germany; m.lengl@tum.de (M.L.); stefan.roehrl@tum.de (S.R.); kldi@tum.de (K.D.); 3 Heinz-Nixdorf-Chair of Biomedical Electronics, TUM School of Computation, Information and Technology, Technical University of Munich, TranslaTUM, 81675 Munich, Germany; christian.klenk@tum.de (C.K.); oliver.hayden@tum.de (O.H.)

**Keywords:** digital holographic microscopy, differential cell count, differential leukocyte count, somatic cell count, dairy cow, immunomonitoring, immunophenotyping, udder health

## Abstract

For several years, the determination of a differential cell count of a raw milk sample has been proposed as a more accurate tool for monitoring the udder health of dairy cows compared with using the absolute somatic cell count. However, the required sample preparation and staining process can be labor- and cost-intensive. Therefore, the aim of our study was to demonstrate the feasibility of analyzing unlabeled blood and milk leukocytes from dairy cows by means of digital holographic microscopy (DHM). For this, we trained three different machine learning methods, i.e., k-Nearest Neighbor, Random Forests, and Support Vector Machine, on sorted leukocyte populations (granulocytes, lymphocytes, and monocytes/macrophages) isolated from blood and milk samples of three dairy cows by using fluorescence-activated cell sorting. Afterward, those classifiers were applied to differentiate unlabeled blood and milk samples analyzed by DHM. A total of 70 blood and 70 milk samples were used. Those samples were collected from five clinically healthy cows at 14-time points within a study period of 26 days. The outcome was compared with the results of the same samples analyzed by flow cytometry and (in the case of blood samples) also to routine analysis in an external laboratory. Moreover, a standard vaccination was used as an immune stimulus during the study to check for changes in cell morphology or cell counts. When applied to isolated leukocytes, Random Forests performed best, with a specificity of 0.93 for blood and 0.84 for milk cells and a sensitivity of 0.90 and 0.81, respectively. Although the results of the three analytical methods differed, it could be demonstrated that a DHM analysis is applicable for blood and milk leukocyte samples with high reliability. Compared with the flow cytometric results, Random Forests showed an MAE of 0.11 (SD = 0.04), an RMSE of 0.13 (SD = 0.14), and an MRE of 1.00 (SD = 1.11) for all blood leukocyte counts and an MAE of 0.20 (SD = 0.11), an RMSE of 0.21 (SD = 0.11) and an MRE of 1.95 (SD = 2.17) for all milk cell populations. Further studies with larger sample sizes and varying immune cell compositions are required to establish method-specific reference ranges.

## Simple Summary

Milk contains immune cells that migrate from the bloodstream into the mammary gland. As this influx increases during an immune reaction, the number of leukocytes is routinely measured for diagnostic purposes in veterinary medicine. In addition to the total number, the individual cell types can be differentiated for a more in-depth analysis. In this study, we demonstrate that digital holographic microscopy (DHM) can be used to identify leukocyte populations in bovine milk and blood without the need for extensive and costly sample preparation steps, such as cell staining. For this, we used different cell types that were isolated from milk and blood samples of dairy cows to train and test machine learning methods. We then applied these methods to analyze unknown milk and blood samples and compared the output with established analytical methods. Although the results varied, our findings show that DHM is a promising and reliable diagnostic tool.

## 1. Introduction

The determination of the somatic cell count (SCC) of a milk sample has long been used to monitor the udder health of dairy cows [1]. For this purpose, the absolute number of body cells contained in a raw milk sample is calculated in order to ascertain the total quantity of leukocytes that have crossed the blood–milk barrier during lactation. In case of an inflammation of the mammary gland (mastitis), a large number of immune cells migrate into the udder tissue and eventually end up in the secreted milk via diapedesis, causing an increase of the SCC. Thus, the SCC value can give a rough estimate to distinguish between a healthy and a diseased udder [2]. However, as the leukocytes in milk consist of different immune cell subtypes with various functions during an immune response [3,4], it can be useful to determine the individual components through a differential cell count (DCC) to gain a deeper insight into the immunological status of the mammary gland. Furthermore, given the fact that milk samples are frequently available and can be taken non-invasively, they are a convenient sample material. Therefore, several DCC-based biomarkers have been proposed in recent years, as extensively reviewed by Farschtschi et al. [5], to assess the health status of a bovine mammary gland. Many of those markers are based on flow cytometry [6,7,8]. However, since staining with fluorochrome-conjugated antibodies and flow cytometric analysis are time-consuming and cost-intensive, new and comparatively simple methods are preferable. Such methods could facilitate the implementation of DCC determination in routine dairy diagnostics, such as the monthly Dairy Herd Improvement (DHI) testing.

Digital holographic microscopy (DHM) is one such promising approach that could help overcome these drawbacks [9,10]. It is a flexible quantitative phase imaging technique that allows researchers to investigate the kinetic and morphological properties of intact single cells free of labeling costs while preserving a high number of cellular characteristics. Moreover, DHM can be combined with a microfluidic system enabling high throughput rates that yield results with thorough statistical validity. In recent years, several promising DHM studies have been presented in various fields of human medicine. Gupta et al. [11] and Vercruysse et al. [12] outlined the possibility of distinguishing different subsets of leukocytes in human blood. In hematology, Ugele et al. [13] and Paidi et al. [14] demonstrated the advantages of holographic cell imaging for the diagnosis and monitoring of human leukemia. Kim et al. [15] could show the versatility of the approach for the screening of hematologic disorders. Regarding immunothrombosis, Klenk et al. [16] and Nishikawa et al. [17] proved that this technology is capable of capturing volatile biomarkers and using them for the prediction of previously barred disease progressions in humans. By taking this a step further and refining the setup for a specific use case, real-time analysis can be achieved [18]. These examples illustrate the flexible application of the DHM technology for hematology and immunothrombosis diagnostics. To the best of our knowledge, comparable approaches in veterinary medicine are still lacking. However, its use in veterinary medicine is especially advisable, as many standardized tests are still very expensive, time-consuming, or even unavailable, and the computer vision-based DHM approach could provide a quick remedy without an extensive sample preparation and staining process.

Thus, the aim of our study was to investigate the feasibility of using high-throughput DHM in combination with machine learning algorithms to analyze unlabeled single leukocytes. For this, we worked with bovine blood samples, as this method has already been successfully applied to human hematology. In addition, we focused on raw milk samples from the same dairy cows in order to transfer the knowledge gained from blood analysis to this complex biofluid and to ascertain whether any changes in milk cell populations were also reflected in blood.

## 2. Methods

### 2.1. Animal Study

This feasibility study was conducted on five clinically healthy Brown Swiss cows between first and third lactation (176–254 days in milk) with a daily milk yield between 23.8 kg and 31.8 kg (for details, see Supplementary Table S1). All cows were kept under optimal conditions in compliance with good agricultural practices at Veitshof, a research station of the TUM School of Life Sciences (Technical University of Munich in Freising, Germany). They were housed in a cubicle housing system with rubber-coated slatted floors and milked twice a day in a 2 × 2 tandem milking parlor (GEA WestfaliaSurge GmbH, Bönen, Germany). All animals had *ad libitum* access to fresh water and were fed on average a daily feed ratio of 18 kg corn silage, 14 kg grass silage, and 1.5 kg hay, supplemented with 1.5 kg high-protein rape and soy extraction meal (deuka Kompopur 404, Deutsche Tiernahrung Cremer, Düsseldorf, Germany) and 190 g mineral mix (Complett Keragen Longlife, Josera, Kleinheubach, Germany). Additionally, 0.5 kg concentrated feed (deuka MK 194-UDP, Deutsche Tiernahrung Cremer, Düsseldorf, Germany) per liter of delivered milk was added to the diet to meet the energy need for the respective performance.

In order to assess if an immune stimulus would influence the cell morphology or the cell counts, all cows were vaccinated during the trial with Bovilis Rotavec Corona (Intervet Deutschland GmbH, Unterschleißheim, Germany). This vaccine contains deactivated strains of the bovine rotavirus (UK-Compton serotype G6 P5), the bovine coronavirus (strain Mebus), and *E. coli* (CN7985 serotype 0101:K99:F41). The vaccination was applied to the healthy cows by a veterinarian as recommended by the manufacturer. All cows were examined at least on every sampling day, and their health status was documented.

**Figure 1:**
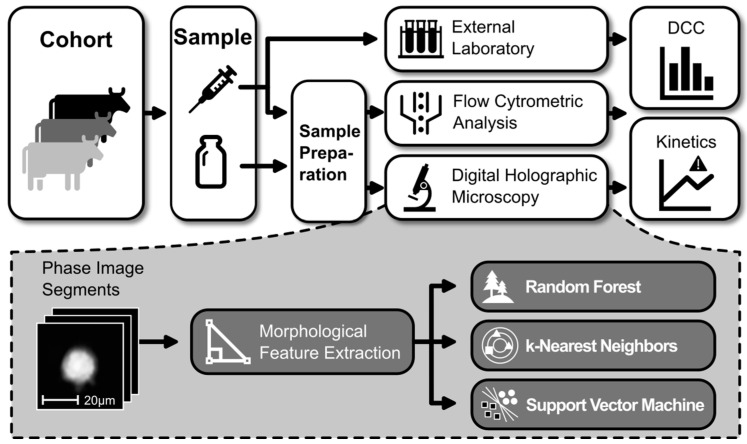
Schematic overview of the workflow.

This whole study was conducted in concordance with the German Animal Welfare Act (TierSchG) and the German regulations on the welfare of animals used for experiments or other scientific purposes (Tierschutz-Versuchstierverordnung, TierSchVersV). The animal study was permitted by the government of Upper Bavaria in Munich, Germany (reference number ROB-55.2-2532.Vet_03-17-70). Figure 1 provides a schematic overview of the workflow and the applied methods.

### 2.2. Sampling and Cell Isolation

**Figure 2:**
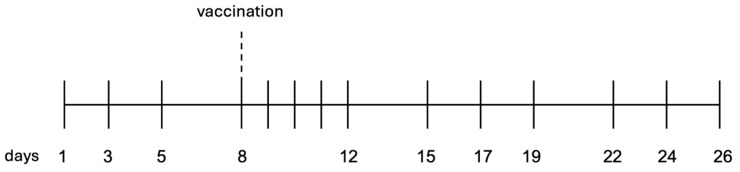
Sampling scheme of blood and milk samples. Blood and milk samples were collected from each of the five cows at 14 time points. All cows were vaccinated on day 8 after sampling.

Blood and milk samples were taken before and after the vaccination, according to the sampling scheme (see Figure 2). Within a total study period of 26 days, four blood and four milk samples were collected from each study animal before the vaccination (i.e., on days 1, 3, 5, and 8) and ten samples each were taken after vaccination (i.e., on days 9, 10, 11, 12, 15, 17, 19, 22, 24, and 26). The respective sampling procedure and the cell isolation protocol were performed as described in detail by Farschtschi, Mattes, Hildebrandt, Chiang, Kirchner, Kliem, and Pfaffl [8]. In brief, fresh milk samples were collected in the milking machine throughout the whole morning milking process. As the samples were brought directly to the laboratory, no preservative was added. The milk samples were centrifuged three times, and the cell pellets were washed after each centrifugation with DPBS (Dulbecco’s Phosphate Buffered Saline, Sigma Aldrich, Co., Saint Louis, MO, USA).

On the same day, the blood samples were obtained from the jugular vein after morning milking. After incubation with an ACK lysis buffer (0.15 M NH_4_Cl, 13 mM KCl, 0.1 mM Na_2_EDTA, pH 7.4, sterile filtered), the samples were centrifuged three times, and the cell pellets were washed with the same lysis buffer.

Subsequently, the isolated milk and blood cell numbers were determined using a TC10 Automated Cell Counter (Bio-Rad Laboratories Inc., Hercules, CA, USA). For the analysis in the digital holographic microscope, 3 × 10^6^ milk or blood cells were transferred into a separate tube and mixed with 1 mL of cold FACS buffer (DPBS with 2% fetal bovine serum (Sigma Aldrich, Co., Saint Louis, MO, USA) and 0.01% NaN_3_). For the flow cytometric analysis, samples with 1 × 10^6^ cells in 1 mL FACS buffer were prepared. Throughout all the aforementioned sample preparation steps, samples and reagents were kept on ice.

The results of these blood and milk samples were later included in the Vaccination Dataset; see below.

### 2.3. Flow Cytometric Analysis

For the flow cytometric (FACS) analysis, milk and blood leukocyte samples were stained with a viability dye and fluorochrome-labeled antibodies (Supplementary Material, Tables S2 and S3) and fixated for overnight storage, summarized in detail by Farschtschi, Mattes, Hildebrandt, Chiang, Kirchner, Kliem, and Pfaffl [8]. On the following day, the samples were analyzed using a BD LSRFortessa flow cytometer (Becton, Dickinson and Company, Franklin Lakes, NJ, USA) and the corresponding BD FACSDiva software v8. Raw FACS data were assessed with FlowJo software v10 (Becton, Dickinson and Company, Franklin Lakes, NJ, USA). In the first step, debris, doublets, and dead cells were excluded. Subsequently, leukocytes (CD45^+^) were differentiated into granulocytes (SSC^high^), monocytes/macrophages (SSC^mid^), and lymphocytes (SSC^low^). For an exemplary gating, see Supplementary Material, Figure S1.

### 2.4. Cell Sorting

To obtain isolated samples of the different leukocyte populations, the blood and milk cells of three of the cows were sorted with a BD FACSAria Fusion flow cytometer (Becton, Dickinson and Company, Franklin Lakes, NJ, USA) and BD FACSDiva software v8. After excluding doublets and dead cells, granulocytes (SSC^high^CD45^+^CD11b^+^) and monocytes/macrophages (SSC^mid^CD45^+^CD14^+^) were selected and separated. In a different run, several lymphocyte subpopulations, i.e., gd T cells (SSC^low^CD45^+^gdTCR^+^), T helper cells (SSC^low^CD45^+^CD4^+^), cytotoxic T cells (SSC^low^CD45^+^CD8^+^), NK cells (SSC^low^CD45^+^CD335^+^) and B cells (SSC^low^CD45^+^CD21^+^), were collected and pooled. A detailed list of the used reagents can be found in the Supplementary Material (Tables S2 and S3).

These isolated blood and milk samples were included in the Isolation Dataset; see below.

### 2.5. Digital Holographic Microscopy (DHM)

Through the interference of an object beam and a reference beam, quantitative phase information of cells can be obtained. Differences in phase shifts within a cell are a result of minor differences in refractive indices and cell heights [19,20]. These phase shifts can later be translated into brighter and darker areas of the image, creating a contrast that allows the differentiation of cells and cell components without the use of expensive antibodies of color staining. In combination with a microfluidic channel, samples can be measured in flow at high throughput rates, drastically reducing the measurement time. Furthermore, the design with four sheath flows ensures that all cells are in the same focal plane so that the risk of occlusion can be neglected. A detailed description of the imaging technique can be found elsewhere [21].

In this work, we used a customized microscope with an SLED for flow cytometry, allowing us to measure single cells with high precision. Although our approach shares most of its properties with common off-axis DHM [20], it is a common-path phase microscopy method using a low-coherence Köhler illumination for parallelized cell imaging at 105 fps. The microfluidic channel is built using a 50 × 500 μm polymethyl methacrylate that employs four sheath flows to center the bloodstream in the focal plane and to avoid contact with the channel walls [13,21]. In our study, we recorded about 10,000 images per measurement with, on average, 5 cells/frame.

Prior to the measurement with the DHM, the blood and milk leukocyte samples were centrifuged (5 min, 500× *g*) and then diluted in 300 mL of a solution containing 99.95% PBS and 0.05% PEO (polyethylene oxide with a molecular weight of 4 × 10^6^ Da, Sigma Aldrich, Co., Saint Louis, MO, USA).

### 2.6. Additional Analysis of Blood Samples by an External Laboratory

In addition, EDTA blood and serum samples were sent to a commercial veterinary laboratory (Laboklin GmbH & Co. KG, Bad Kissingen, Germany) for the determination of routine biomarkers and a differential blood count. Standard differential blood cell counts were performed with an ADVIA 2120i (Siemens Healthcare GmbH, Erlangen, Germany).

### 2.7. Data Processing

#### 2.7.1. Pre-Processing

The DHM setup used provides images with a resolution of 512 × 384 pixels using a 40× NA 0.55 objective, where each pixel value represents the phase shift at that position. As each of these images can contain multiple cells, they need to be preprocessed to obtain usable single-cell patches. Initially, we eliminate possible background noise and artifacts caused by the microfluidic channel by subtracting the median taken from a set of 1000 images. This step is feasible because the channel environment can be assumed to be static throughout the whole recording session. Following that, we utilize threshold segmentation, setting a threshold of 0.8 to identify individual cells. For each detected cell covering an area of at least 357 µm² (equivalent to 30 pixels), we extract an image patch of 96 × 96 pixels. Cells smaller than this threshold are usually remnants of the isolation process. The phase shift values stored in each pixel of the image patch serve as input for the subsequent feature extraction.

#### 2.7.2. Feature Extraction

Different populations of milk and blood leukocytes have varying morphological appearances. We computed a set of 24 hand-crafted cellular features (Supplementary Material, Table S4) that describe, e.g., the optical volume, the contrast, the size, or the optical height of the cells [13,14,22]. Later, this catalog of 24 cellular features serves as input for the classification models.

#### 2.7.3. Dataset

During our experiments, we worked with two datasets. The Isolation Dataset contains the sorted cells of three cows (compare section “Cell Sorting”) and is used to teach the models the existing cellular differences. The inherent imbalance in this dataset, caused by the biologically uneven distribution of cell types, underscores the need for applying an appropriate sampling technique to avoid overfitting the later trained models. As the number of samples in the minority classes is quite small, under-sampling would result in too small datasets. To avoid overfitting on the few existing samples, we applied a synthetic minority over-sampling technique [23] that interpolates additional samples based on a nearest-neighbor approach. This results in 51,995 samples per class for blood and 9713 samples per class for milk. The Vaccination Dataset contains the samples of all cows for all measurement days (compare Figure 2).

#### 2.7.4. Classification

Three different standard classifiers, i.e., k-Nearest Neighbor, Random Forests, and Support Vector Machine, were evaluated for the analysis of the extracted features. In order to keep the complexity low and to provide reasonable explainability, the focus was put on classical machine learning methods instead of black box neural networks. In comparison to applying convolutional neural networks directly on the images, this approach allows all decisions made to be traced back to human-understandable cell characteristics. An overview of the successful application of the selected classifiers was provided by Poostchi et al. [24]. The k-Nearest Neighbor (kNN) [25] algorithm takes the k-nearest neighbors of an unknown sample into account and selects the label that is most represented among these neighbors. Random Forests (RFs) [26] combine the output of several decision trees [27] to assign a label. Each tree starts with all samples, and at each node, a single feature is used to split the samples into two groups. After several nodes, the tree ends up with pure groups that belong to one label. The Support Vector Machine (SVM) [28] approach tries to find a hyperplane in a multidimensional space that best separates the given samples. The optimal separation is reached when the distance between the hyperplane and the samples is maximized.

#### 2.7.5. Evaluation

Within our experiments we use several metrics for evaluating the predictive models regarding the different aspects of model performance. Accuracy measures the proportion of correct predictions out of all predictions made, giving an overall sense of model correctness. Sensitivity is the ability of a model to correctly identify positive cases, making it crucial for contexts such as medical diagnoses where detecting true positives is important. Specificity measures the ability to correctly identify negative cases, which is essential when minimizing false positives is critical. On the error side, Mean Absolute Error (MAE) calculates the average magnitude of errors in a model’s predictions without considering direction, giving a straightforward measure of accuracy. Mean Relative Error (MRE) is similar but expresses errors as a percentage, which is useful for comparing performance across different scales. Finally, Root Mean Square Error (RMSE) is the square root of the average squared errors, placing higher emphasis on larger errors, making it effective in identifying substantial prediction deviations. Each subsection of Section 2.8 names the metrics that are used for the individual experiment, respectively.

### 2.8. Experiments

#### 2.8.1. Evaluation of the Different Classifiers and Their Hyperparameters

As a first step, we conducted an exhaustive grid search to identify the classifier and parameter combination best suited for these given data. The combinations explored during this search are detailed in the Supplementary Material, Table S5. The experiment is performed on the Isolation Dataset. To assess and compare the final models, 30% of these data are reserved for testing purposes. The remaining 70% of samples undergo a 5-fold cross-validation process, as described by Kohavi [29], where each parameter combination is trained five times. During each iteration, one subset is designated for validation while the others are used for training. The comparison is evaluated in terms of accuracy, specificity, and sensitivity [30]. After finding the best parameters for all classifiers, they are re-fitted using all data to ensure maximal usage of available information.

#### 2.8.2. Classifying Unknown Test Subjects

Approaching a scenario closer to real-world application, we evaluate the efficacy of our developed methods in classifying blood and milk samples from unknown individuals. Using the Isolation Dataset*,* we divide these data such that always two cows are used for training, and the remaining one is used for testing. The performance is once again evaluated in terms of accuracy, specificity, and sensitivity.

#### 2.8.3. Prediction of Unlabeled Cells and Comparison of These DHM Results to Results of Flow Cytometric Analysis and to Results of External Laboratory

In this experiment, we employ the three classifiers from above with their respective best parameter sets to analyze the Vaccination Dataset. For every measurement date and each cow, we predict the type for each cell and determine their proportions within the sample. To evaluate the results, we compare the shares with the values from the flow cytometric analysis, both visually and quantitively, regarding the MAE, MRE, and RMSE. In addition, the results of the blood analysis carried out by the external laboratory are likewise compared with the blood results of the Vaccination Dataset. Moreover, the cell count progressions over time were examined for changes before and after vaccinations.

## 3. Results

### 3.1. Visualization of Analyzed Cells

**Figure 3:**
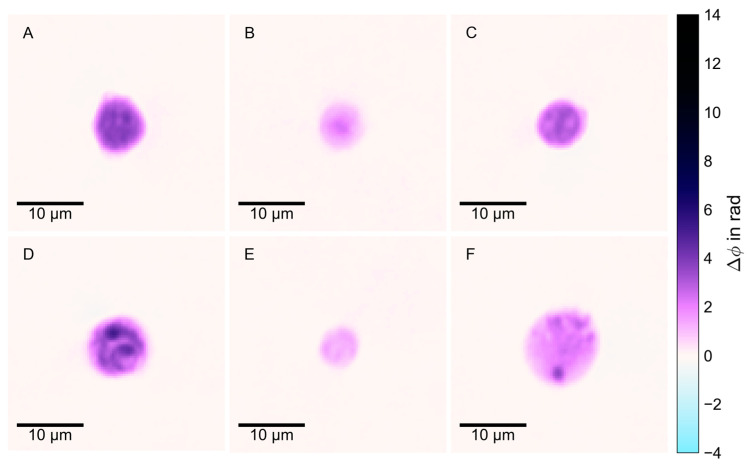
Representative false-color phase images of different populations of unlabeled leukocytes, analyzed in DHM. (**A**) Blood granulocyte; (**B**) Blood lymphocyte; (**C**) Blood monocyte; (**D**) Milk granulocyte; (**E**) Milk lymphocyte; (**F**) Milk macrophage.

The detected cells in blood and milk samples were displayed as phase images, in which the degree of phase shift (measured in rad) between the cell and the reference beam was visualized. For illustrative purposes, false-color images were created for a selection of the analyzed cell populations by assigning a color to certain phase shift values. For exemplary false-color phase images, see Figure 3.

**Figure 4:**
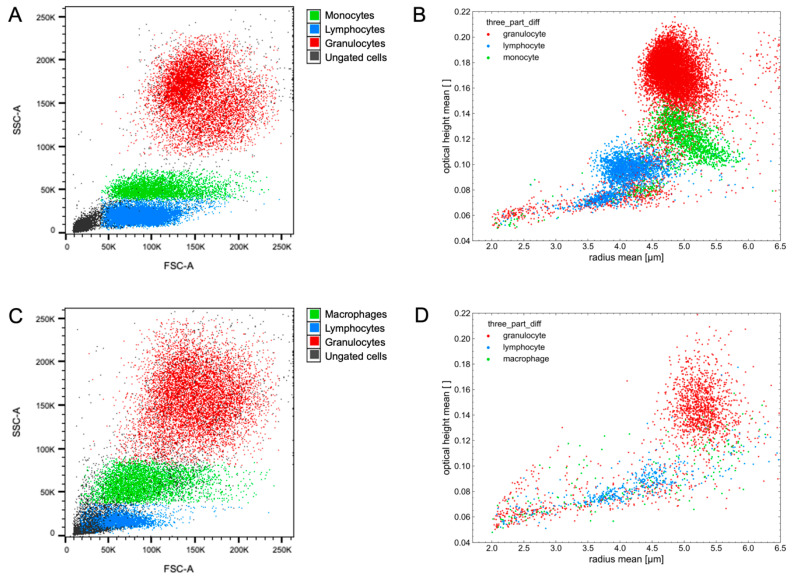
Exemplary scatter plots of flow cytometric and digital holographic microscopy analyses. (**A**) Blood leukocyte populations analyzed by FACS; (**B**) Blood leukocyte populations analyzed by DHM; (**C**) Milk leukocyte populations analyzed by FACS; (**D**) Milk leukocyte populations analyzed by DHM.

To highlight the discriminative power of the selected features, they can be visualized in scatter plots. These plots show a similar pattern to those obtained from flow cytometric analysis, where the cell types form dense clusters that can be distinguished from one another. For exemplary scatter plots of both methods, see Figure 4. Alternatively, each feature can be presented in a kernel density estimate (KDE) plot to exhibit the differences of the separate cell types. For KDE plots of all 24 features, see Supplementary Figures S2–S7.

### 3.2. Efficacy of Trained Classifiers to Identify Sorted Cells of the Test Set

Of the three classifiers tested on the Isolation Dataset, RFs performed best. The sorted blood cells included in the test set could be identified with a specificity of 0.93 and a sensitivity of 0.90. For the milk cells, a specificity of 0.84 and a sensitivity of 0.81 could be achieved. Using SVM, a specificity of 0.92 and a sensitivity of 0.91 were reached for blood cells, and a specificity of 0.80 and a sensitivity of 0.82 for milk cells. With kNN, blood cells could be identified with a specificity and a sensitivity of 0.90 and 0.87, respectively, and milk cells with a specificity of 0.82 and a sensitivity of 0.76. For details of the different populations, see Supplementary Material, Tables S6–S11.

**Figure 5:**
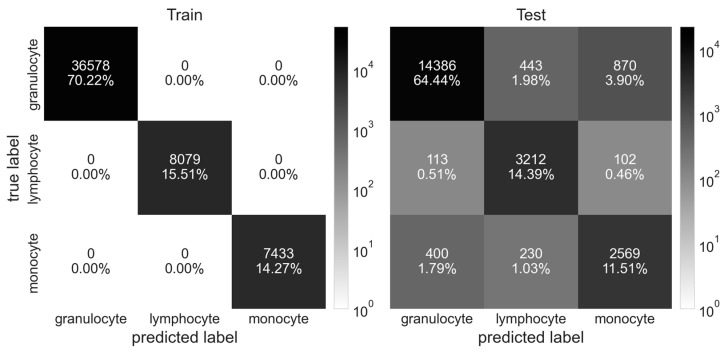
Confusion matrix showing the results of Random Forest classification of sorted blood cells.

**Figure 6:**
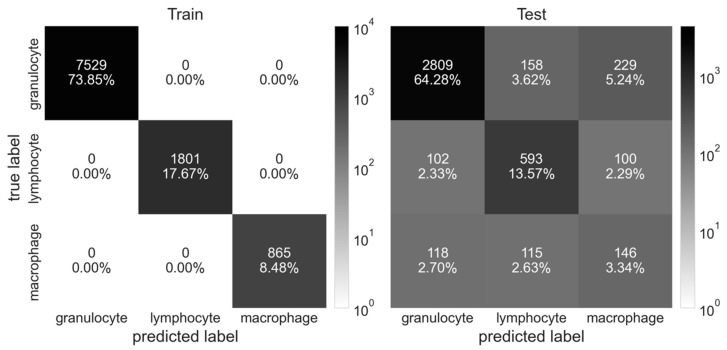
Confusion matrix showing the results of Random Forest classification of sorted milk cells.

To visualize the performance of the different classifiers, confusion matrices were plotted as exemplarily shown for RF in Figure 5 for blood cells and in Figure 6 for milk cells.

### 3.3. Outcome of the Classification of an Unknown Test Subject

When the three selected classifiers were trained on the Isolation Dataset of two cows and tested on the Isolation Dataset of the respective third cow, all classifiers performed similarly well, with RF being slightly superior. In detail, RF reached a mean specificity in blood of 0.82 (SD = 0.2) and milk of 0.78 (SD = 0.04), with a mean sensitivity of 0.84 (SD = 0.04) and 0.78 (SD = 0.05), respectively. SVM identified the blood cells in the test set with a mean specificity of 0.82 (SD = 0.01) and a mean sensitivity of 0.84 (SD = 0.04). For milk cells, SVM was performed with a mean specificity of 0.73 (SD = 0.07) and a mean sensitivity of 0.79 (SD = 0.04). In this 3-fold cross-validation, kNN achieved a mean specificity of 0.82 (SD = 0.03) in blood and 0.76 (SD = 0.04) in milk. The mean sensitivity in blood was 0.81 (SD = 0.04) and 0.73 (SD = 0.05) in milk. For further details, please see Supplementary Material, Tables S12–S17.

### 3.4. Comparison of DHM Results to Results Obtained with Flow Cytometry and Blood Counts of the External Laboratory

**Figure 7:**
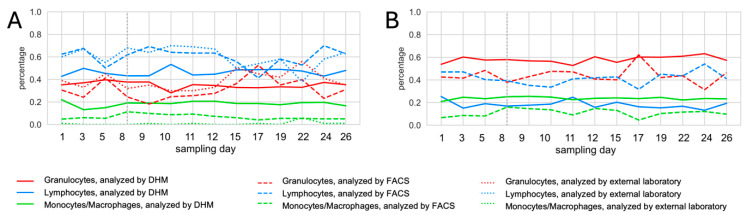
Cell count progression over time, DHM results obtained using k-Nearest Neighbor. (**A**) Blood cells of cow #963, analyzed by DHM, FACS and external laboratory; (**B**) Milk cells of cow #963, analyzed by DHM and FACS.

The values obtained by DHM were not overall consistent compared with the results of the FACS analysis and the differential blood counts analyzed by the external laboratory. Displayed cell count progressions over time for all analysis methods can be found in Figure 7, exemplarily for one cow. For all cell counts, refer to Supplementary Figures S18–S20. The overall results obtained by RF for all blood cell types showed an MAE of 0.11 (SD = 0.04), an RMSE of 0.13 (SD = 0.14), and an MRE of 1.00 (SD = 1.11) when compared with flow cytometric data. When focusing on the different blood cell populations, monocytes were those with the highest MRE of 2.48 and the highest cow individual differences, which resulted in an SD of 0.67. For blood granulocytes and blood lymphocytes, the MRE levels were considerably lower, i.e., 0.29 and 0.23, respectively, with less variance between individuals, i.e., SD values of 0.15 and 0.06, respectively. The differences in DHM results (determined with RF) and flow cytometric results were more pronounced for milk cells. The comparison for all cell types revealed an MAE of 0.20 (SD = 0.11), an RMSE of 0.21 (SD = 0.11), and an MRE of 1.95 (SD = 2.17). Here, macrophages were the cell population with the highest MRE (3.99) and the highest variation between subjects (SD = 2.38). Milk lymphocytes could be identified with an MRE of 1.27 (SD = 1.31). In milk, the best agreement between the two analysis methods was achieved for granulocytes, resulting in an MRE of 0.58 and the lowest cow-individual differences (SD = 0.58). A similar pattern was found when comparing the results of kNN and SVM with the outcome of the flow cytometric analysis of blood and milk cells. In addition to the flow cytometric results, the DHM blood values were compared with the differential blood count obtained at the external laboratory. The overall comparison of all blood cells resulted in an MAE of 0.14 (SD = 0.06), an RMSE of 0.15 (SD = 0.06), and an MRE of 0.20 (SD = 0.07). The best agreement was documented for granulocytes (MRE = 0.16) with the lowest SD value of 0.02. For granulocyte counts, an MRE of 0.25 and an SD value of 0.07 could be achieved. For results of kNN and SVM, as well as the detailed results of all cows, please see Supplementary Material, Tables S18–S20.

Regarding the impact of vaccination on cell counts, the respective KDE plots of the morphological features, shown in Supplementary Figures S8–S13, do not display any visually observable differences between cells sampled before and after the vaccination. Since these plots did not indicate any noticeable differences, no additional statistical validation was performed. Similarly, there were no visually significant changes in the percentages of immune cell populations documented in blood and milk samples taken after the cows had been vaccinated.

## 4. Discussion

Several studies have proposed the use of a DCC determined from raw milk samples as an advanced tool for monitoring the udder health status of dairy cows [31,32,33] and potentially even their general health status [8]; however, all previous approaches require some degree of sample preparation and staining, which can be time-consuming and costly. In this regard, the use of DHM can offer a clear advantage as no labeling is required. Moreover, all detected particles can be observed directly during the DHM measurement as grayscale phase images. In addition, once these data have been processed, each segmented single-cell image can be displayed, e.g., as false-color phase images, and thereby examined. This allows visual verification of the automatic classification performed by the analysis software. In contrast, using flow cytometry, false positive events can only be ascertained by means of control samples, such as isotype or fluorescence minus one control [34].

When the three classifiers were tested with the Isolation Dataset, all performed with a satisfactory outcome, especially for blood granulocytes and blood lymphocytes. However, the results for milk cells were less accurate than those for blood cells. This could have been caused by the lower number of milk cells contained in the Isolation Dataset. Furthermore, the purification of a raw milk sample is more challenging than the isolation of leukocytes from sterile blood samples and could have led to residual debris in the Isolation Dataset. Particularly for milk macrophages, a reliable identification was difficult. This may be due to the very limited number of macrophages available in the training set or the variable appearance of this cell type in milk [35]. Moreover, the sorting of blood monocytes could have been more precise if they had been labeled with antibodies directed against CD172a instead of CD14, as this marker was described by Grandoni et al. [36] as superior in identifying monocytes. To improve the accuracy of the analysis of the acquired DHM data, convolutional neural networks could be applied as an alternative to classical machine learning methods. Such deep learning algorithms are inspired by biological nervous systems comparable to the human brain and have a high potential to differentiate and recognize image patterns [37,38].

The results of the DHM analysis deviated from data acquired by means of flow cytometry analysis and by the external laboratory. These noted inconsistencies between the applied methods could be due to the different preparation methods or—in the case of the external laboratory—the absence of cell isolation steps. It is known that sample preparation, such as the use of a lysis buffer [39], can influence cell morphology. In addition, repeated washing steps may result in the loss of cells. It is, therefore, advisable to define distinct reference ranges for each method. In future studies, the milk sample preparation steps should be optimized to minimize alterations or cell loss and to facilitate the application of DHM in routine diagnostics. Given that other milk constituents, such as fat globules, can also be detected by DHM [40], it should be feasible to differentiate leukocytes in unprocessed whole milk samples.

No significant differences could be detected in the distribution of morphological features before and after vaccination. Thus, it can be concluded that the determination of a DCC can be reliably performed based on the morphology of leukocyte populations, even in different health states. However, variations between the individual cows were observed. Therefore, studies with larger sample sizes would be necessary to train the classifiers more accurately and to establish reference ranges for the different leukocyte populations.

Given the great potential of this easy-to-apply, high-throughput method and the fact that no cell staining is required, it seems worth pursuing this innovative approach further. One possible area of application could be the monthly DHI testing, in which a DHM-based DCC could be implemented to monitor udder health as an extension of the established SCC. Furthermore, the development of a point-of-care test could be considered, for example, to support mastitis diagnosis or selective dry cow therapy.

## 5. Conclusions

In this first feasibility study, the applicability of DHM measurements to identify bovine leukocyte populations was demonstrated for both blood and milk cells. The different machine learning methods were trained and successfully tested on isolated blood and milk leukocyte populations, with milk being the more challenging medium to work with. However, when compared with established cell analysis methods, the results were not overall consistent. To address this, further studies with more test subjects and samples of various breeds are needed to overcome the high inter-animal variation and to establish method-specific reference ranges.

## Data Availability

Data can be made available by the corresponding author upon request.

## Supplementary Materials

The following supporting information can be downloaded at: https://www.mdpi.com/article/10.3390/ani14213156/s1, Figure S1: Exemplary gating strategy; Figure S2: Density of morphological features of different blood leukocyte populations. A: Contrast. B: Correlation. C: Dissimilarity. D: Energy. E: Entropy. F: Homogeneity. G: Area. H: Aspect Ratio; Figure S3: Density of morphological features of different blood leukocyte populations. I: Biconcavity. K: Circularity. L: Density. M: Discocyte Error. N: Mass Center Shift. O: Optical Height Mass. P: Optical Height Min. Q: Optical Height Mean; Figure S4: Density of morphological features of different blood leukocyte populations. R: Optical Height Std. S: Radius Max. T: Radius Min. U: Radius Mean. V: Radius Std. W: Solidity. X: Steepness. Y: Volume; Figure S5: Density of morphological features of different milk leukocyte populations. A: Contrast. B: Correlation. C: Dissimilarity. D: Energy. E: Entropy. F: Homogeneity. G: Area. H: Aspect Ratio; Figure S6: Density of morphological features of different milk leukocyte populations. I: Biconcavity. K: Circularity. L: Density. M: Discocyte Error. N: Mass Center Shift. O: Optical Height Mass. P: Optical Height Min. Q: Optical Height Mean; Figure S7: Density of morphological features of different milk leukocyte populations. R: Optical Height Std. S: Radius Max. T: Radius Min. U: Radius Mean. V: Radius Std. W: Solidity. X: Steepness. Y: Volume; Figure S8: Density of different morphological features of blood cells before and after vaccination. A: Contrast. B: Correlation. C: Dissimilarity. D: Energy. E: Entropy. F: Homogeneity. G: Area. H: Aspect Ratio; Figure S9: Density of different morphological features of blood cells before and after vaccination. I: Biconcavity. K: Circularity. L: Density. M: Discocyte Error. N: Mass Center Shift. O: Optical Height Mass. P: Optical Height Min. Q: Optical Height Mean; Figure S10: Density of different morphological features of blood cells before and after vaccination. R: Optical Height Std. S: Radius Max. T: Radius Min. U: Radius Mean. V: Radius Std. W: Solidity. X: Steepness. Y: Volume; Figure S11: Density of different morphological features of milk cells before and after vaccination. A: Contrast. B: Correlation. C: Dissimilarity. D: Energy. E: Entropy. F: Homogeneity. G: Area. H: Aspect Ratio; Figure S12: Density of different morphological features of milk cells before and after vaccination. I: Biconcavity. K: Circularity. L: Density. M: Discocyte Error. N: Mass Center Shift. O: Optical Height Mass. P: Optical Height Min. Q: Optical Height Mean; Figure S13: Density of different morphological features of blood cells before and after vaccination. R: Optical Height Std. S: Radius Max. T: Radius Min. U: Radius Mean. V: Radius Std. W: Solidity. X: Steepness. Y: Volume; Figure S14: Cell count progressions over time, DHM results analyzed using k-Nearest Neighbor classification; Figure S15: Cell count progressions over time, DHM results analyzed using Random Forest classification; Figure S16: Cell count progressions over time, DHM results analyzed using Support Vector Machine classification; Table S1: Detailed information about the cows used in this study Table S2: Concentration of viability dye; Table S3: Concentrations of antibodies; Table S4: Descriptions of features; Table S5: Combinations explored for each classifier; Table S6: Specificity of trained classifiers to identify sorted blood cells of test set; Table S7: Sensitivity of trained classifiers to identify sorted blood cells of test set; Table S8: Precision of trained classifiers to identify sorted blood cells of test set; Table S9: Specificity of trained classifiers to identify sorted milk cells of test set; Table S10: Sensitivity of trained classifiers to identify sorted milk cells of test set; Table S11: Precision of trained classifiers to identify sorted milk cells of test set; Table S12: Outcome of classification of blood cells of unknown test subject using k-Nearest Neighbor classification; Table S13: Outcome of classification of milk cells of unknown test subject using k-Nearest Neighbor classification; Table S14: Outcome of classification of blood cells of unknown test subject using Random Forest classification; Table S15: Outcome of classification of milk cells of unknown test subject using Random Forest classification; Table S16: Outcome of classification of blood cells of unknown test subject using Support Vector Machine classification; Table S17: Outcome of classification of milk cells of unknown test subject using Support Vector Machine classification; Table S18: Comparison of DHM results to FACS results for blood cells; Table S19: Comparison of DHM results to FACS results for milk cells; Table S20: Comparison of DHM results to results of external laboratory for blood cells.

## References

[1] Adkins P.R.F., Middleton J.R. (2018). Methods for Diagnosing Mastitis. Vet. Clin. N. Am. Food Anim. Pr. Pract..

[2] Schukken Y.H., Wilson D.J., Welcome F., Garrison-Tikofsky L., Gonzalez R.N. (2003). Monitoring udder health and milk quality using somatic cell counts. Vet. Res..

[3] Sordillo L.M. (2018). Mammary Gland Immunobiology and Resistance to Mastitis. Vet. Clin. N. Am. Food Anim. Pr. Pract..

[4] Oviedo-Boyso J., Valdez-Alarcon J.J., Cajero-Juarez M., Ochoa-Zarzosa A., Lopez-Meza J.E., Bravo-Patino A., Baizabal-Aguirre V.M. (2007). Innate immune response of bovine mammary gland to pathogenic bacteria responsible for mastitis. J. Infect..

[5] Farschtschi S., Mattes M., Pfaffl M.W. (2022). Advantages and Challenges of Differential Immune Cell Count Determination in Blood and Milk for Monitoring the Health and Well-Being of Dairy Cows. Vet. Sci..

[6] De Matteis G., Grandoni F., Scata M.C., Catillo G., Moioli B., Buttazzoni L. (2020). Flow Cytometry-Detected Immunological Markers and on Farm Recorded Parameters in Composite Cow Milk as Related to Udder Health Status. Vet. Sci..

[7] Souza F.N., Blagitz M.G., Batista C.F., Takano P.V., Gargano R.G., Diniz S.A., Silva M.X., Ferronatto J.A., Santos K.R., Heinemann M.B. (2020). Immune response in nonspecific mastitis: What can it tell us?. J. Dairy. Sci..

[8] Farschtschi S., Mattes M., Hildebrandt A., Chiang D., Kirchner B., Kliem H., Pfaffl M.W. (2021). Development of an advanced flow cytometry based high-resolution immunophenotyping method to benchmark early immune response in dairy cows. Sci. Rep..

[9] Jo Y., Cho H., Lee S.Y., Choi G., Kim G., Min H.-s., Park Y. (2019). Quantitative Phase Imaging and Artificial Intelligence: A Review. IEEE J. Sel. Top. Quantum Electron..

[10] Nguyen T.L., Pradeep S., Judson-Torres R.L., Reed J., Teitell M.A., Zangle T.A. (2022). Quantitative Phase Imaging: Recent Advances and Expanding Potential in Biomedicine. ACS Nano.

[11] Gupta R.K., Chen M., Malcolm G.P.A., Hempler N., Dholakia K., Powis S.J. (2019). Label-free optical hemogram of granulocytes enhanced by artificial neural networks. Opt. Express.

[12] Vercruysse D., Dusa A., Stahl R., Vanmeerbeeck G., de Wijs K., Liu C., Prodanov D., Peumans P., Lagae L. (2015). Three-part differential of unlabeled leukocytes with a compact lens-free imaging flow cytometer. Lab. Chip..

[13] Ugele M., Weniger M., Stanzel M., Bassler M., Krause S.W., Friedrich O., Hayden O., Richter L. (2018). Label-Free High-Throughput Leukemia Detection by Holographic Microscopy. Adv. Sci..

[14] Paidi S.K., Raj P., Bordett R., Zhang C., Karandikar S.H., Pandey R., Barman I. (2021). Raman and quantitative phase imaging allow morpho-molecular recognition of malignancy and stages of B-cell acute lymphoblastic leukemia. Biosens. Bioelectron..

[15] Kim G., Jo Y., Cho H., Min H.S., Park Y. (2019). Learning-based screening of hematologic disorders using quantitative phase imaging of individual red blood cells. Biosens. Bioelectron..

[16] Klenk C., Erber J., Fresacher D., Rohrl S., Lengl M., Heim D., Irl H., Schlegel M., Haller B., Lahmer T. (2023). Platelet aggregates detected using quantitative phase imaging associate with COVID-19 severity. Commun. Med..

[17] Nishikawa M., Kanno H., Zhou Y., Xiao T.H., Suzuki T., Ibayashi Y., Harmon J., Takizawa S., Hiramatsu K., Nitta N. (2021). Massive image-based single-cell profiling reveals high levels of circulating platelet aggregates in patients with COVID-19. Nat. Commun..

[18] Nissim N., Dudaie M., Barnea I., Shaked N.T. (2021). Real-Time Stain-Free Classification of Cancer Cells and Blood Cells Using Interferometric Phase Microscopy and Machine Learning. Cytom. A.

[19] Kim M.K. (2010). Principles and techniques of digital holographic microscopy. J. Photonics Energy.

[20] Dubois F., Yourassowsky C. (2015). Off-Axis Interferometer. US Patent.

[21] Klenk C., Fresacher D., Röhrl S., Heim D., Lengl M., Schumann S., Knopp M., Diepold K., Holdenrieder S., Hayden O. (2023). Measurement of Platelet Aggregation in Ageing Samples and After in-Vitro Activation. Proceedings of the 16th International Joint Conference on Biomedical Engineering Systems and Technologies, BIOSTEC 2023.

[22] Pham H.V., Bhaduri B., Tangella K., Best-Popescu C., Popescu G. (2013). Real time blood testing using quantitative phase imaging. PLoS ONE.

[23] Chawla N.V., Bowyer K.W., Hall L.O., Kegelmeyer W.P. (2002). SMOTE: Synthetic Minority Over-sampling Technique. J. Of. Artif. Intell. Res..

[24] Poostchi M., Silamut K., Maude R.J., Jaeger S., Thoma G. (2018). Image analysis and machine learning for detecting malaria. Transl. Res..

[25] Cover T.M., Hart P.E. (1967). Nearest Neighbor Pattern Classification. IEEE Trans. Inf. Theory.

[26] Breiman L. (2001). Random Forests. Mach. Learn..

[27] Breiman L. (1984). Classification and Regression Trees.

[28] Cortes C., Vapnik V. (1995). Support-vector networks. Mach. Learn..

[29] Kohavi R. A study of cross-validation and bootstrap for accuracy estimation and model selection. Proceedings of the 14th international joint conference on Artificial intelligence.

[30] Monaghan T.F., Rahman S.N., Agudelo C.W., Wein A.J., Lazar J.M., Everaert K., Dmochowski R.R. (2021). Foundational Statistical Principles in Medical Research: Sensitivity, Specificity, Positive Predictive Value, and Negative Predictive Value. Medicina.

[31] Rivas A.L., Quimby F.A., Blue J., Coksaygan O. (2001). Longitudinal evaluation of bovine mammary gland health status by somatic cell counting, flow cytometry, and cytology. J. Vet. Diagn. Invest..

[32] Schwarz D., Kleinhans S., Witzel G., Stuckler P., Reith F., Dano S. (2023). Usefulness of the total and differential somatic cell count based udder health group concept for evaluating herd management practices and udder health in dairy herds. Prev. Vet. Med..

[33] Pilla R., Malvisi M., Snel G.G., Schwarz D., Konig S., Czerny C.P., Piccinini R. (2013). Differential cell count as an alternative method to diagnose dairy cow mastitis. J. Dairy. Sci..

[34] Cossarizza A., Chang H.-D., Radbruch A., Akdis M., Andrä I., Annunziato F., Bacher P., Barnaba V., Battistini L., Bauer W.M. (2017). Guidelines for the use of flow cytometry and cell sorting in immunological studies. Eur. J. Immunol..

[35] Sarikaya H., Prgomet C., Pfaffl M.W., Bruckmaier R.M. (2004). Differentiation of leukocytes in bovine milk. Milchwissenschaft.

[36] Grandoni F., Scata M.C., Martucciello A., De Carlo E., De Matteis G., Hussen J. (2021). Comprehensive phenotyping of peripheral blood monocytes in healthy bovine. Cytom. A.

[37] Lindsay G.W. (2021). Convolutional Neural Networks as a Model of the Visual System: Past, Present, and Future. J. Cogn. Neurosci..

[38] LeCun Y., Bengio Y., Hinton G. (2015). Deep learning. Nature.

[39] Klenk C., Heim D., Ugele M., Hayden O. (2019). Impact of sample preparation on holographic imaging of leukocytes. Opt. Eng..

[40] Cheong F.C., Xiao K., Grier D.G. (2009). Technical note: Characterizing individual milk fat globules with holographic video microscopy. J. Dairy. Sci..

