# Supplementary material for: Digital Holographic Microscopy in Veterinary Medicine—A Feasibility Study to Analyze Label-Free Leukocytes in Blood and Milk of Dairy Cows
Source: Animals (Basel). 2024 Nov 3;14(21):3156. doi: 10.3390/ani14213156 (PMC11544890; doi:10.3390/ani14213156)
Supplement: Supplementary file 1 [file animals-14-03156-s001.zip › animals-3214388-supplementary.pdf]

Supplementary Figures

Figure S1. **Exemplary gating strategy.** A: Blood cells, exclusion of debris; B: Blood cells, doublet discrimination; C: Blood cells, determination of viability; D: Blood cells, determination of CD45+ cells; E: Blood cells, differentiation of leukocyte populations; F: Milk cells, exclusion of debris; G: Milk cells, doublet discrimination; H: Milk cells, determination of viability; I: Milk cells, determination of CD45+ cells; J: Milk cells, differentiation of leukocyte populations.

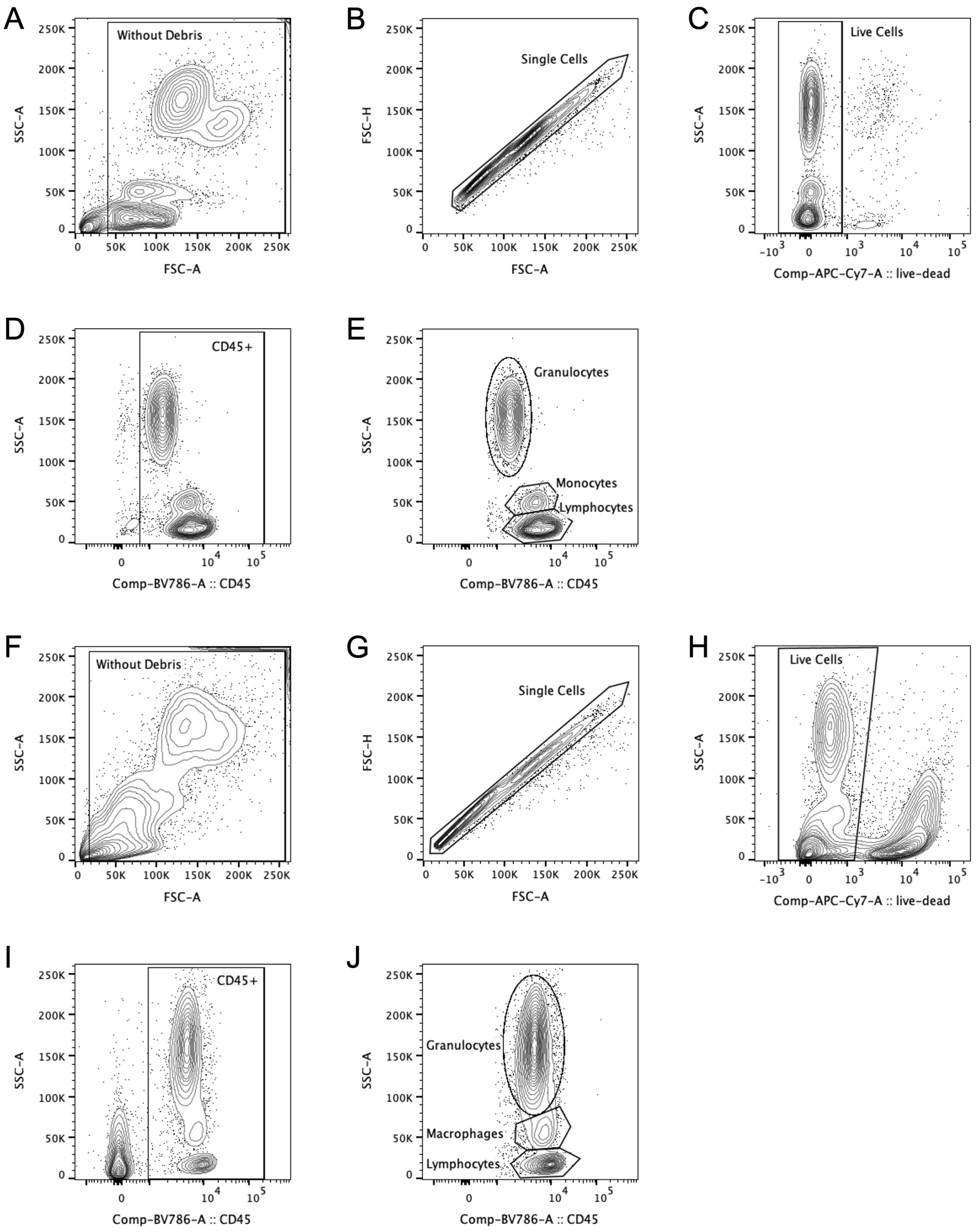

Figure S2. **Density of morphological features of different blood leukocyte populations.** A: Contrast. B: Correlation. C: Dissimilarity. D: Energy. E: Entropy. F: Homogeneity. G: Area. H: Aspect Ratio.

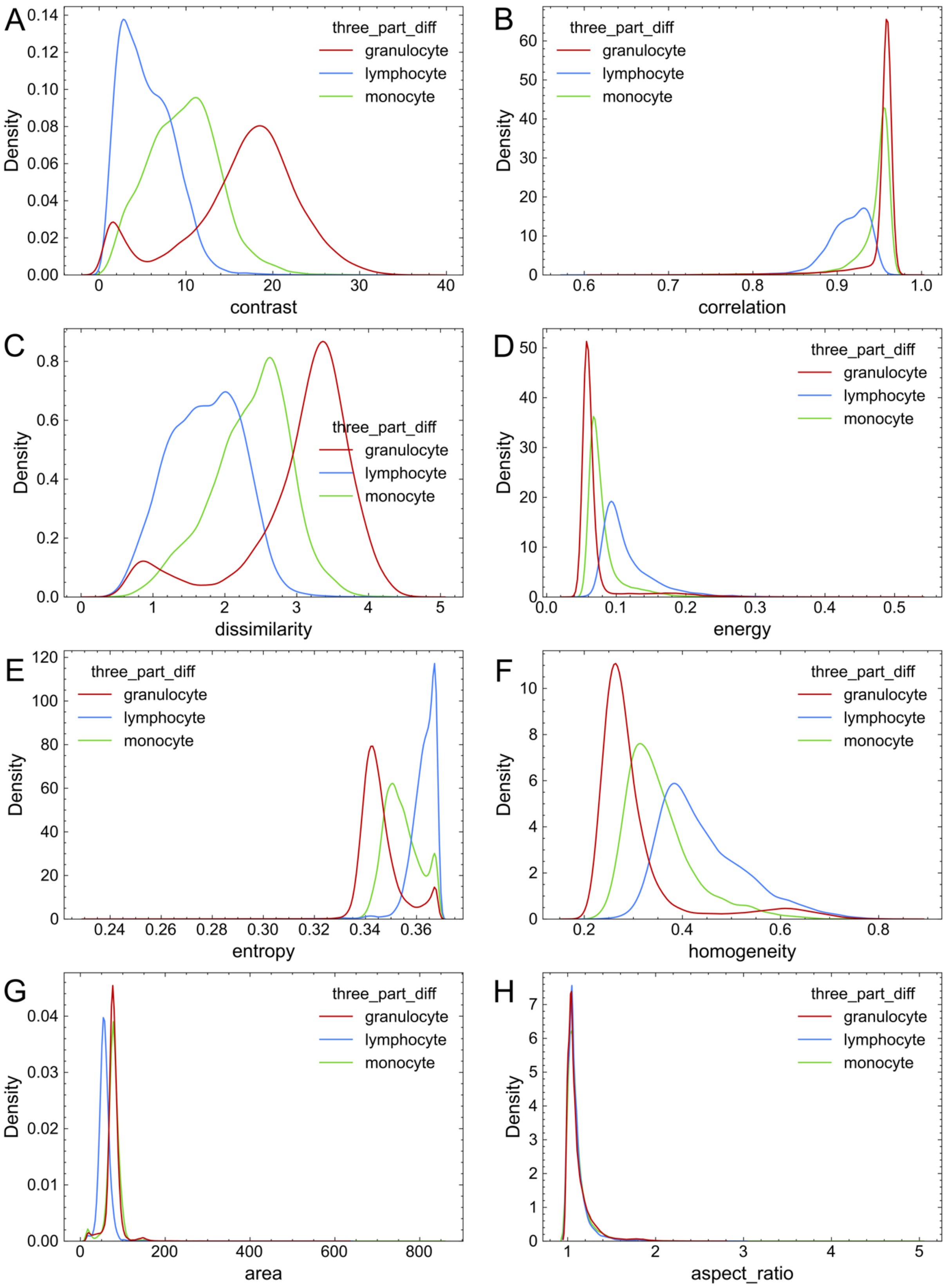

Figure S3. **Density of morphological features of different blood leukocyte populations.** I: Biconcavity. K: Circularity. L: Density. M: Discocyte Error. N: Mass Center Shift. O: Optical Height Mass. P: Optical Height Min. Q: Optical Height Mean.

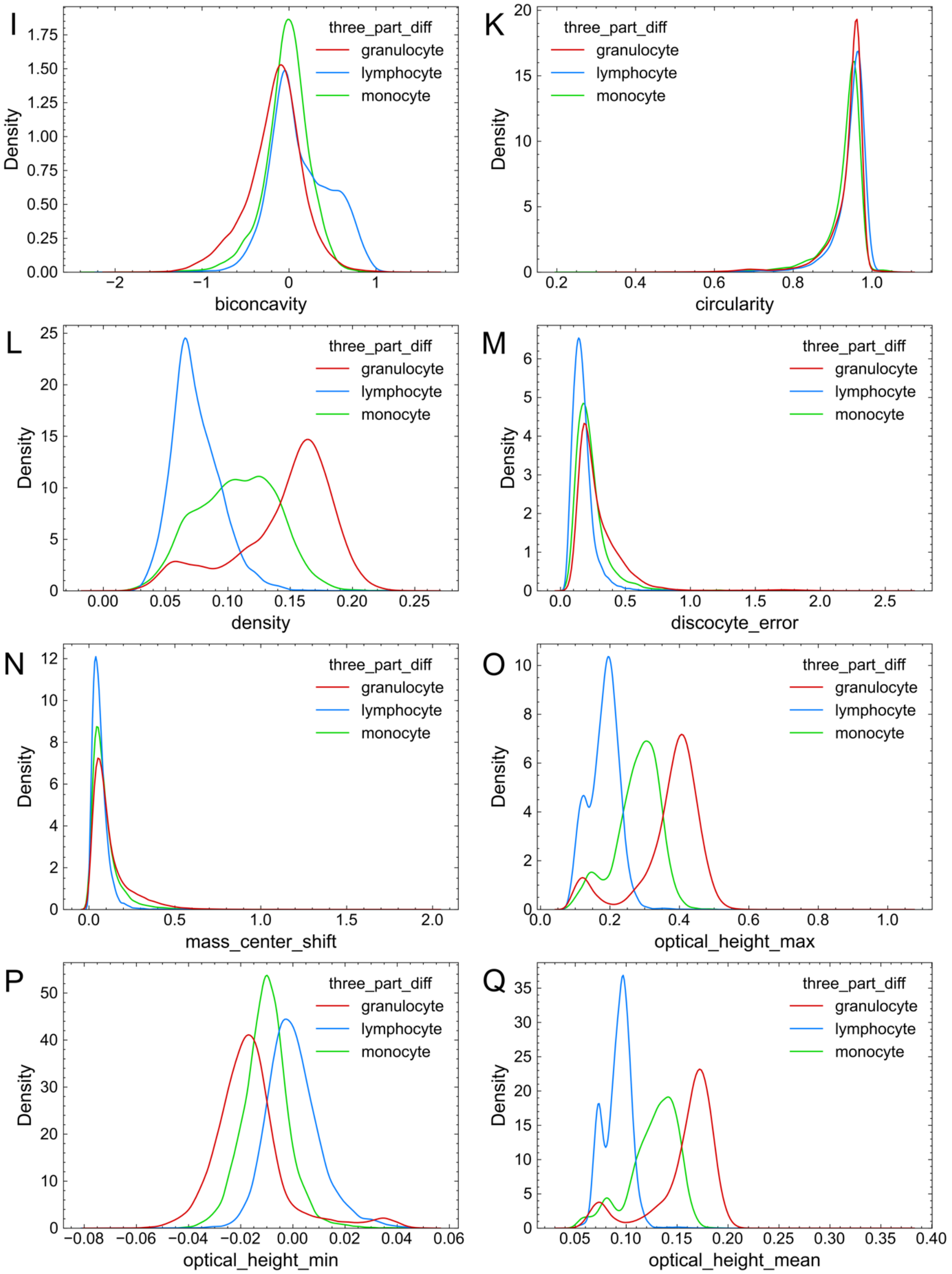

Figure S4. **Density of morphological features of different blood leukocyte populations.** R: Optical Height Std. S: Radius Max. T: Radius Min. U: Radius Mean. V: Radius Std. W: Solidity. X: Steepness. Y: Volume.

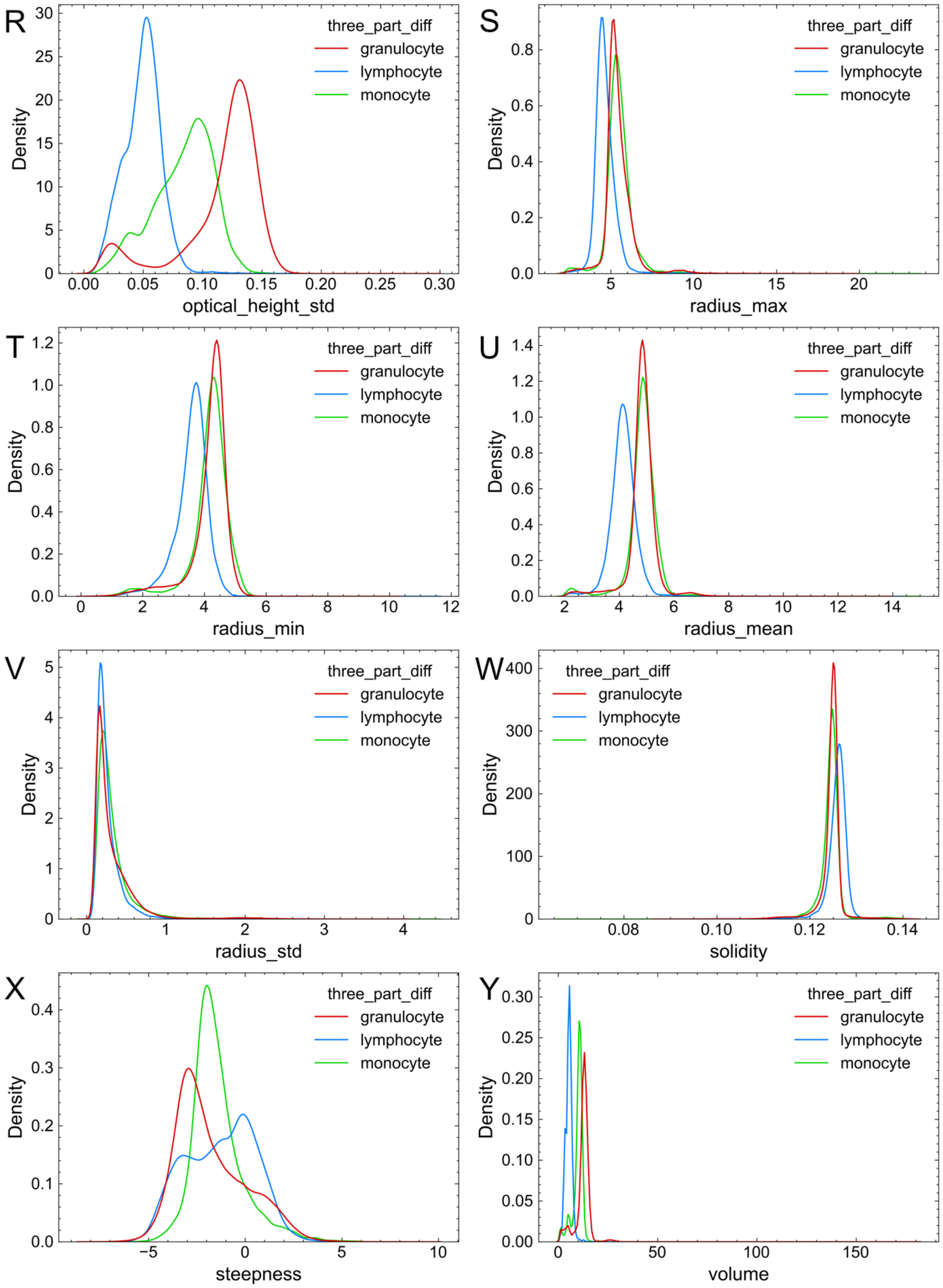

Figure S5. **Density of morphological features of different milk leukocyte populations.** A: Contrast. B: Correlation. C: Dissimilarity. D: Energy. E: Entropy. F: Homogeneity. G: Area. H: Aspect Ratio.

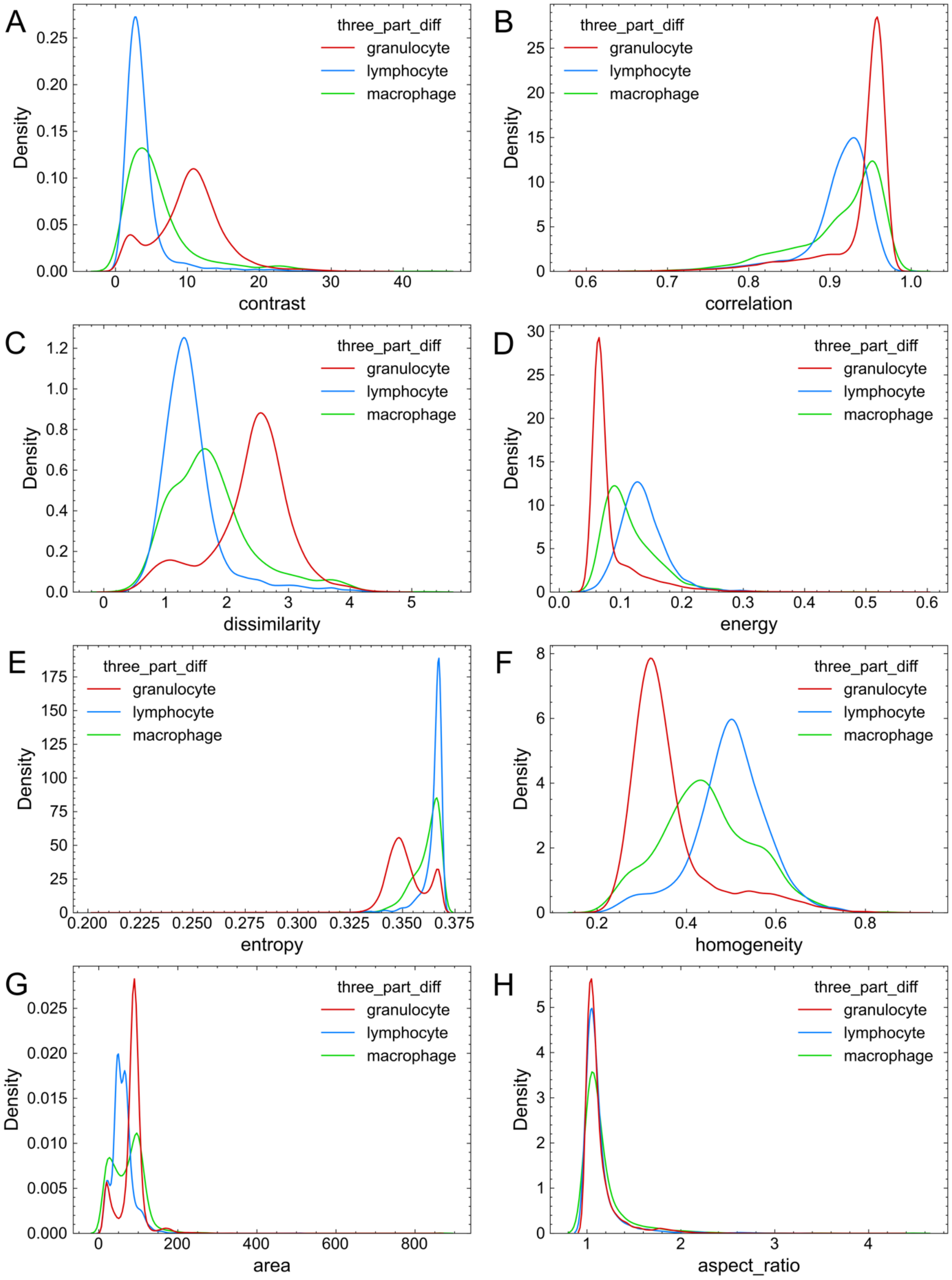

Figure S6. **Density of morphological features of different milk leukocyte populations.** I: Biconcavity. K: Circularity. L: Density. M: Discocyte Error. N: Mass Center Shift. O: Optical Height Mass. P: Optical Height Min. Q: Optical Height Mean.

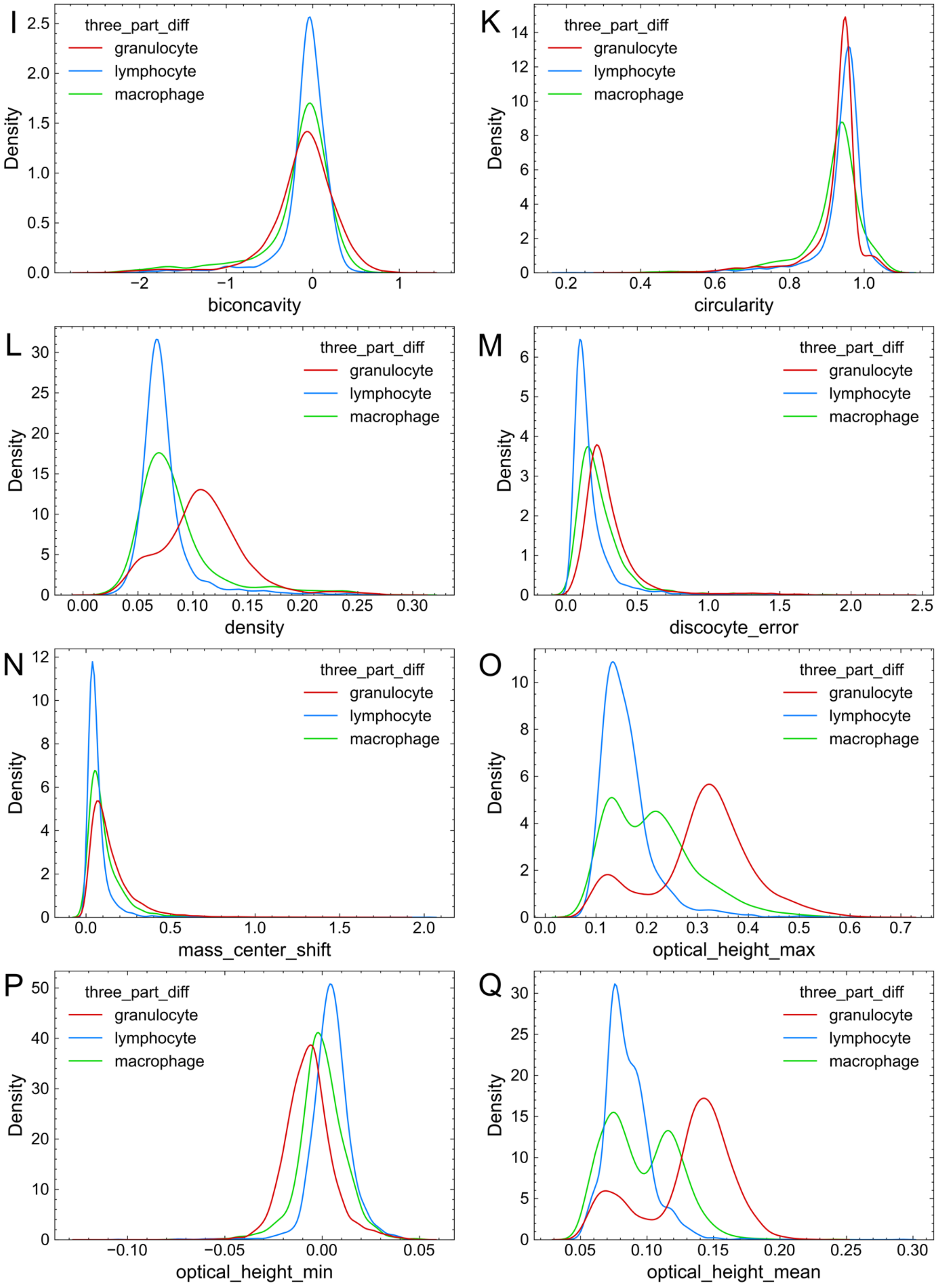

Figure S7. **Density of morphological features of different milk leukocyte populations.** R: Optical Height Std. S: Radius Max. T: Radius Min. U: Radius Mean. V: Radius Std. W: Solidity. X: Steepness. Y: Volume.

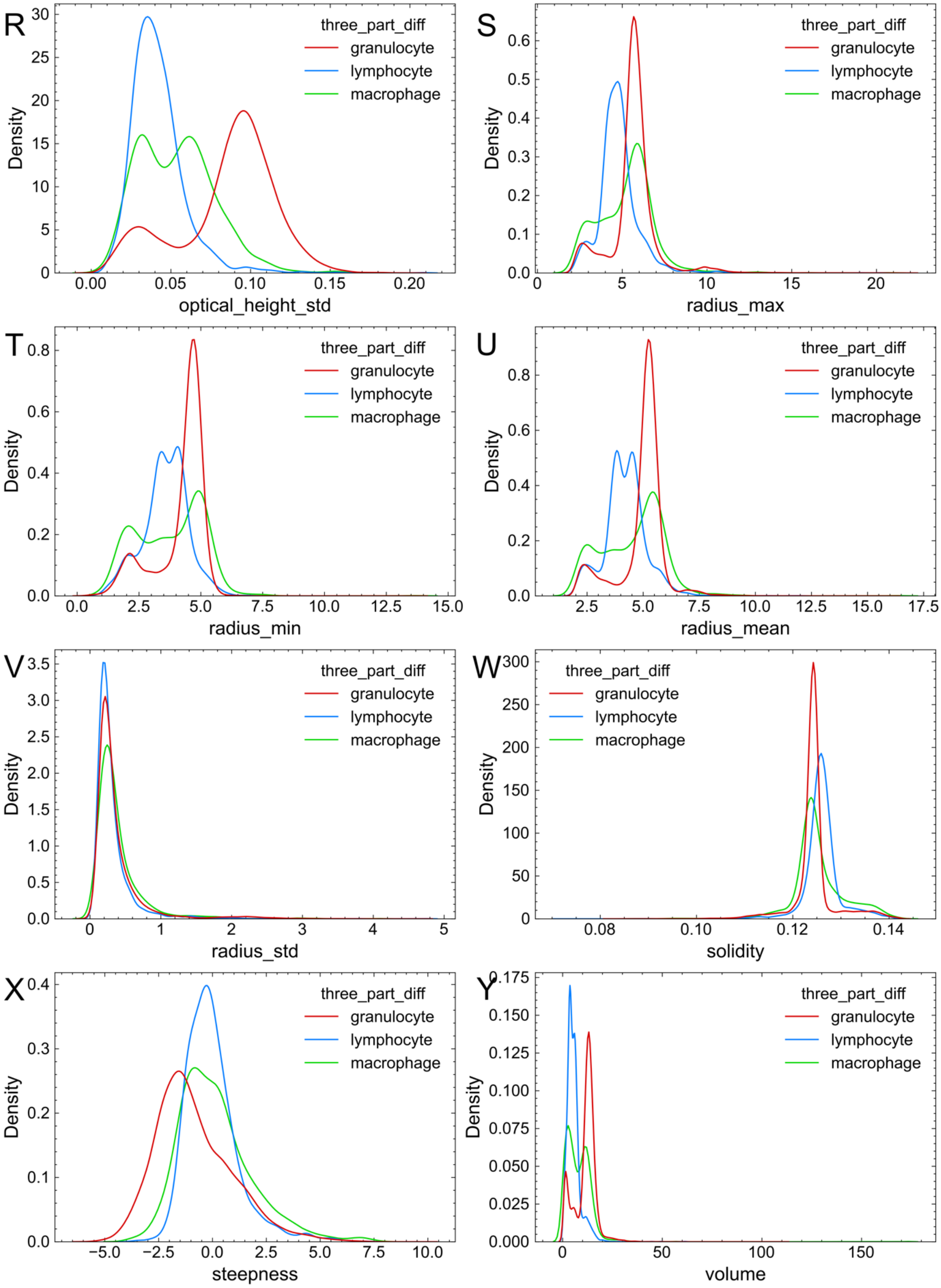

Figure S8. **Density of different morphological features of blood cells before and after vaccination.** A: Contrast. B: Correlation. C: Dissimilarity. D: Energy. E: Entropy. F: Homogeneity. G: Area. H: Aspect Ratio.

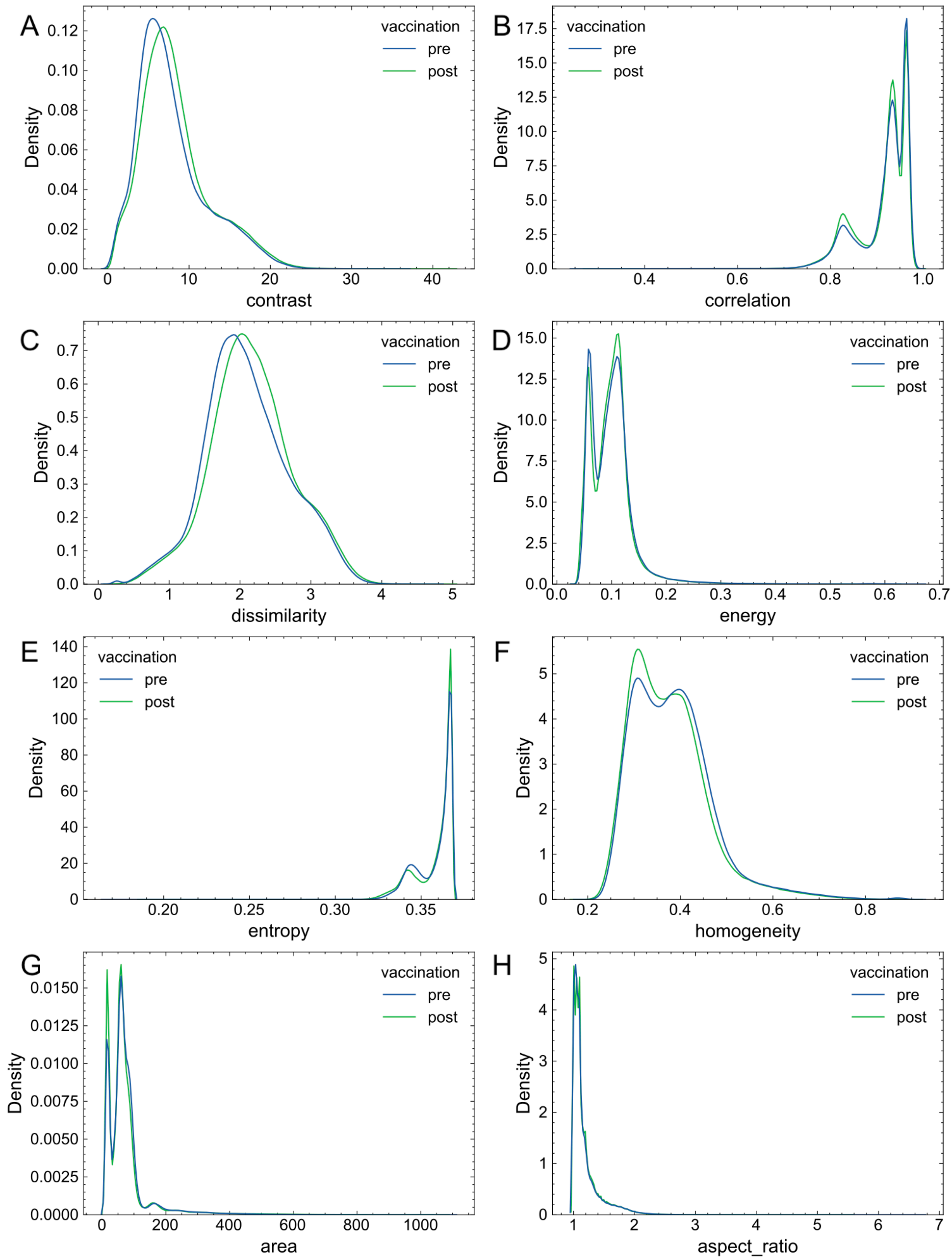

Figure S9. **Density of different morphological features of blood cells before and after vaccination.** I: Biconcavity. K: Circularity. L: Density. M: Discocyte Error. N: Mass Center Shift. O: Optical Height Mass. P: Optical Height Min. Q: Optical Height Mean.

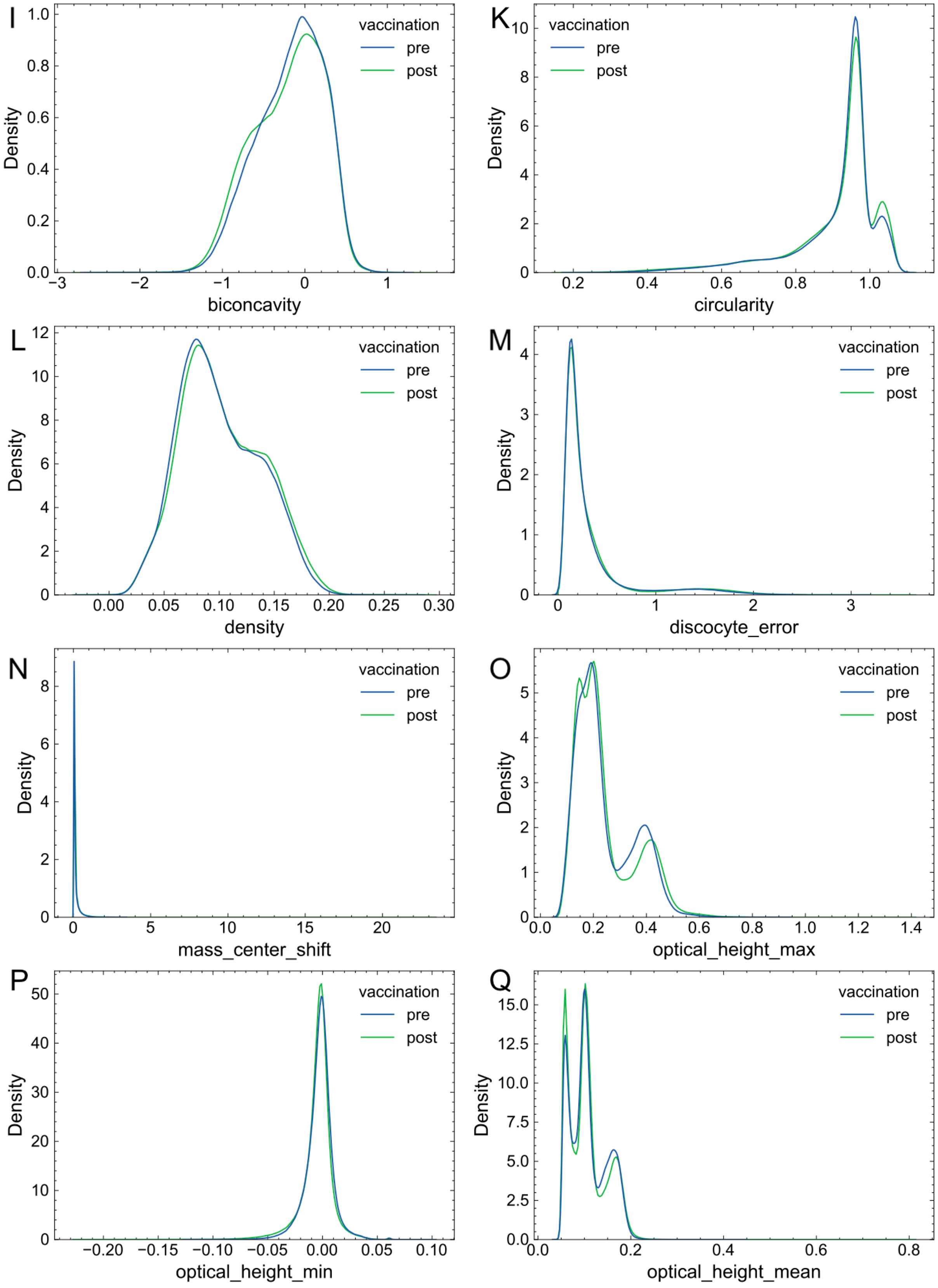

Figure S10. **Density of different morphological features of blood cells before and after vaccination.** R: Optical Height Std. S: Radius Max. T: Radius Min. U: Radius Mean. V: Radius Std. W: Solidity. X: Steepness. Y: Volume.

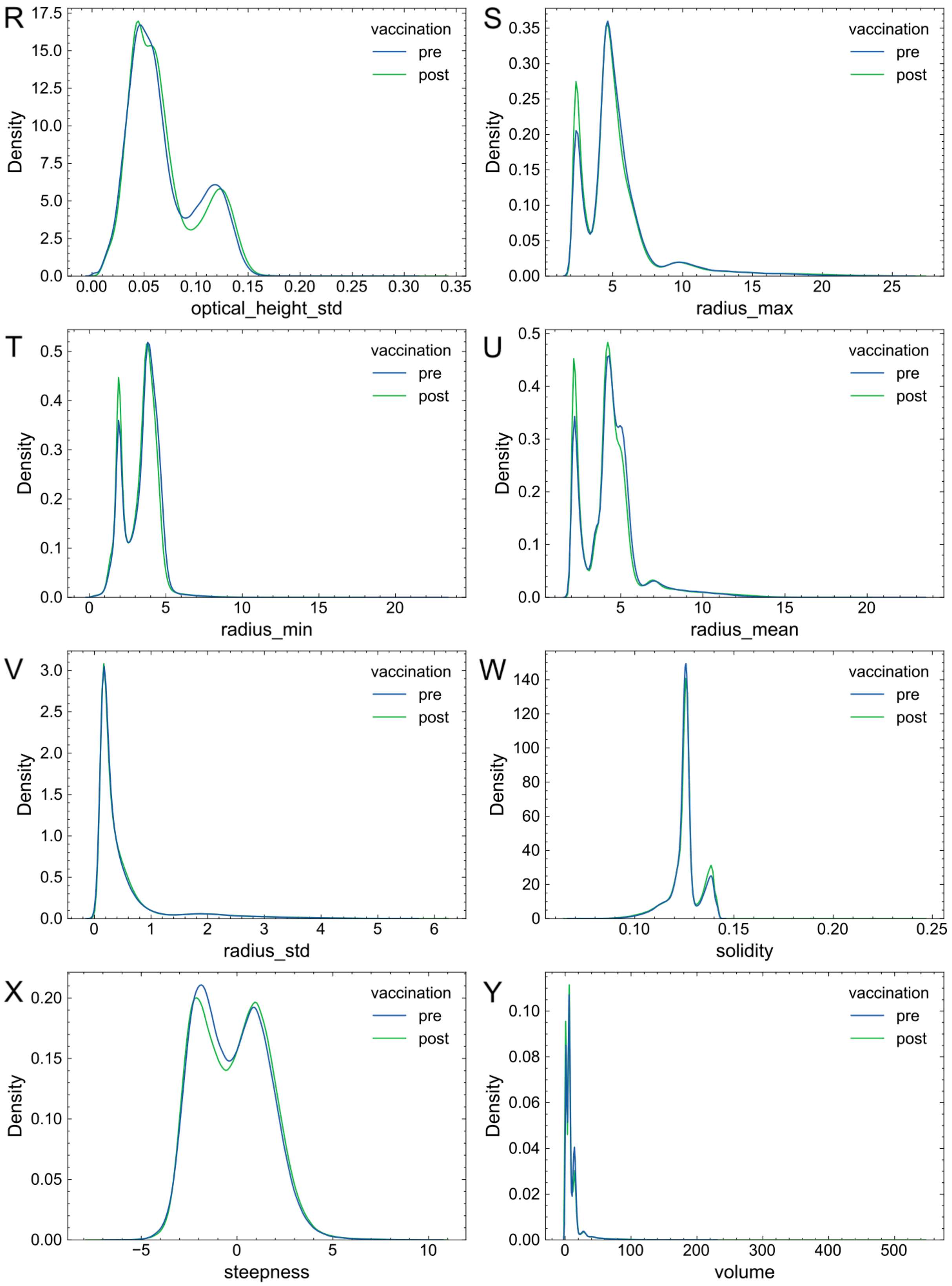

Figure S11. **Density of different morphological features of milk cells before and after vaccination.** A: Contrast. B: Correlation. C: Dissimilarity. D: Energy. E: Entropy. F: Homogeneity. G: Area. H: Aspect Ratio.

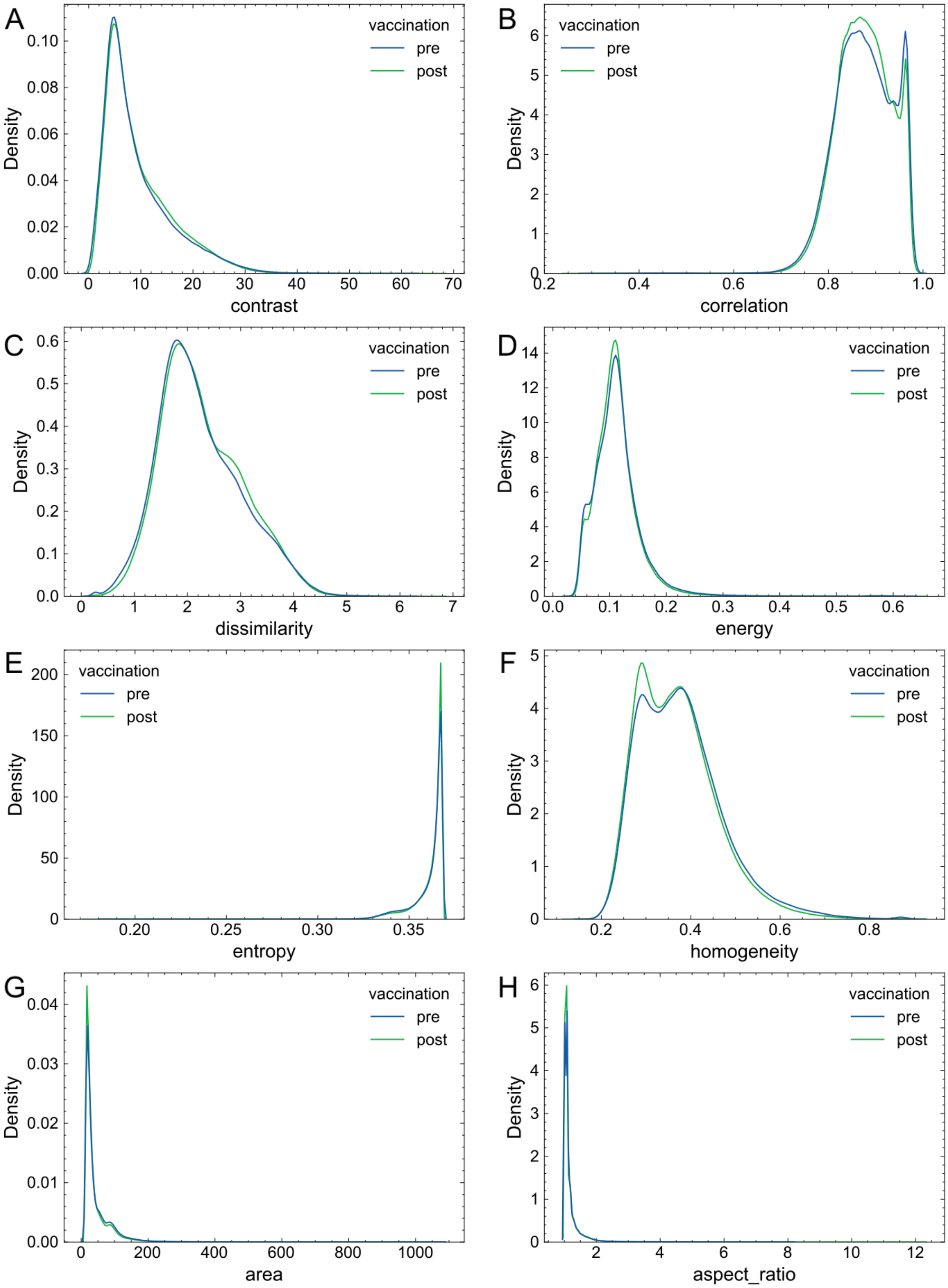

Figure S12. **Density of different morphological features of milk cells before and after vaccination.** I: Biconcavity. K: Circularity. L: Density. M: Discocyte Error. N: Mass Center Shift. O: Optical Height Mass. P: Optical Height Min. Q: Optical Height Mean.

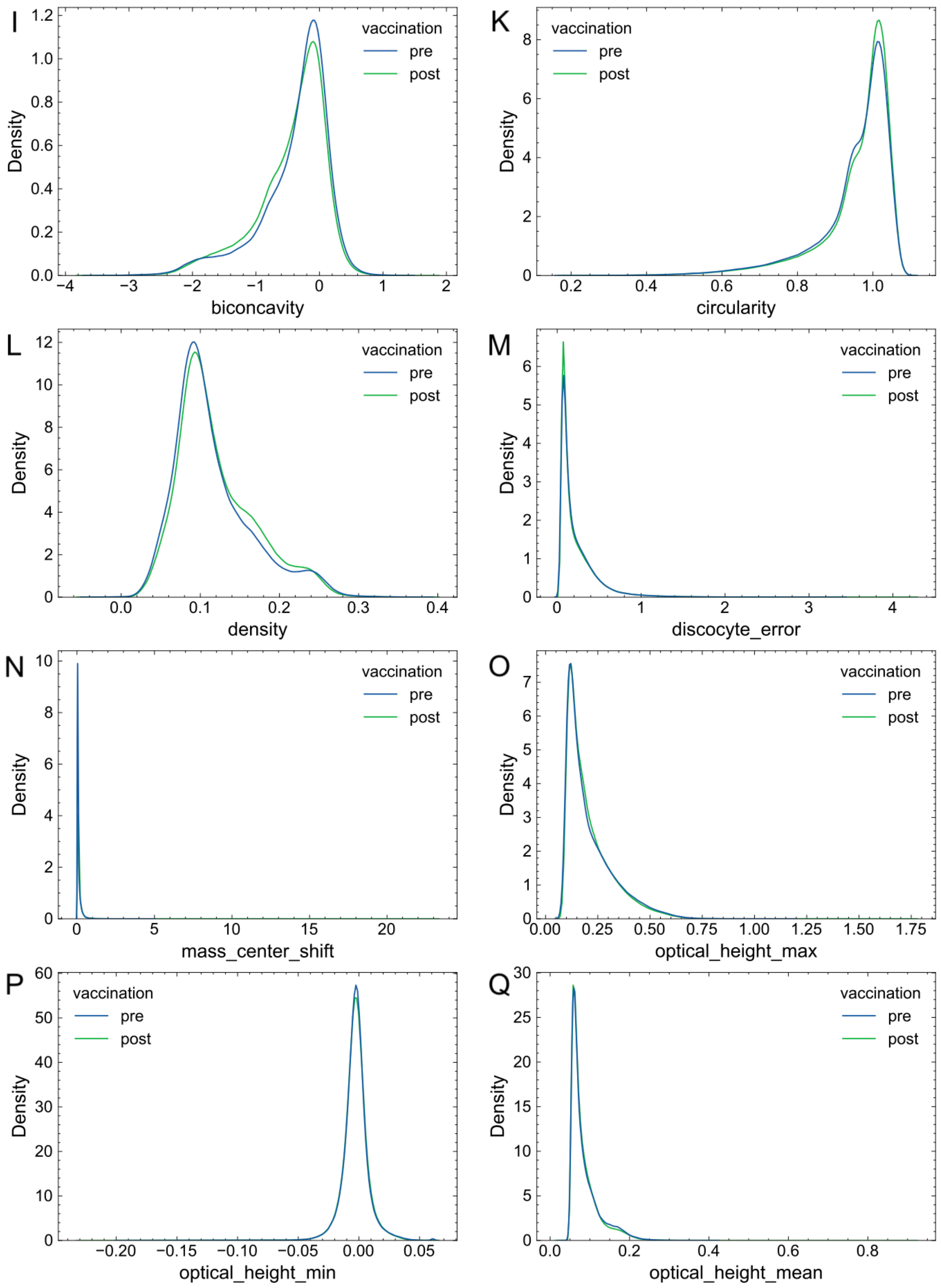

Figure S13. **Density of different morphological features of blood cells before and after vaccination.** R: Optical Height Std. S: Radius Max. T: Radius Min. U: Radius Mean. V: Radius Std. W: Solidity. X: Steepness. Y: Volume.

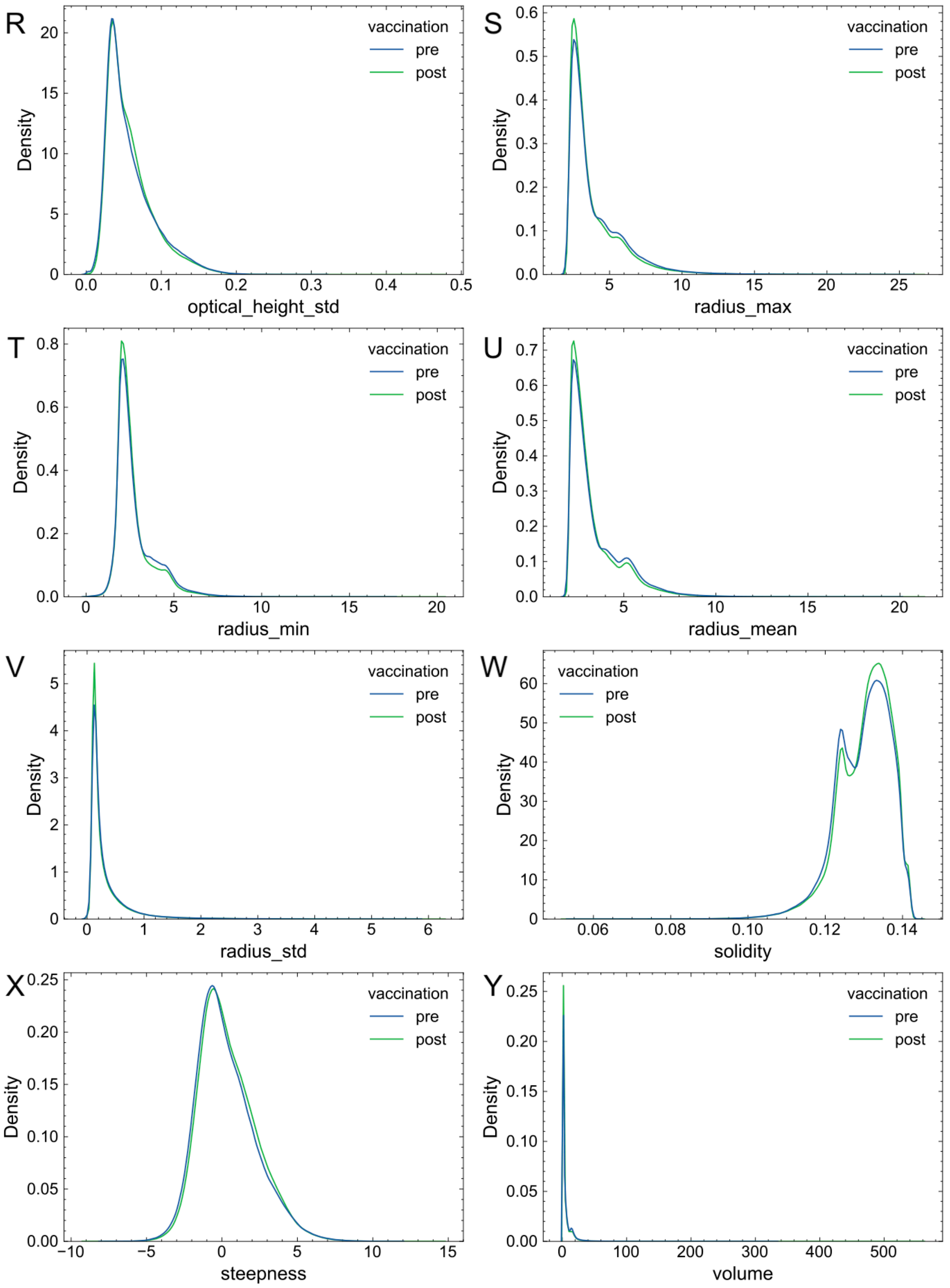

Figure S14. **Cell count progressions over time, DHM results analyzed using k-Nearest Neighbor classification.** A: Blood cells, cow #909. B: Milk cells, cow #909. C: Blood cells, cow #957. D: Milk cells, cow #957. E: Blood cells, cow #963. F: Milk cells, cow #963. G: Blood cells, cow #965. H: Milk cells, cow #965. I: Blood cells, cow #966. K: Milk cells, cow #966.

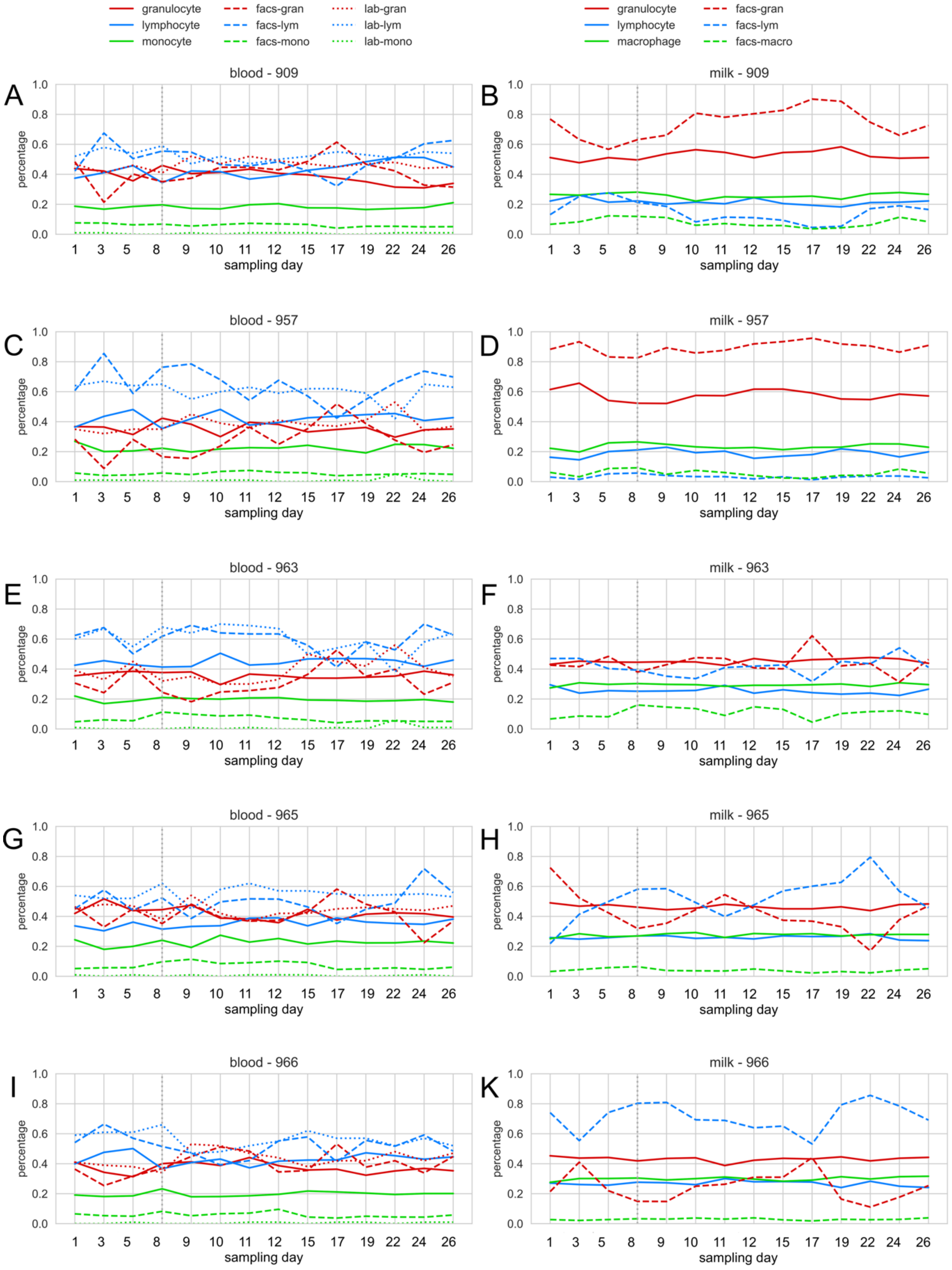

Figure S15. **Cell count progressions over time, DHM results analyzed using Random Forest classification.** A: Blood cells, cow #909. B: Milk cells, cow #909. C: Blood cells, cow #957. D: Milk cells, cow #957. E: Blood cells, cow #963. F: Milk cells, cow #963. G: Blood cells, cow #965. H: Milk cells, cow #965. I: Blood cells, cow #966. K: Milk cells, cow #966.

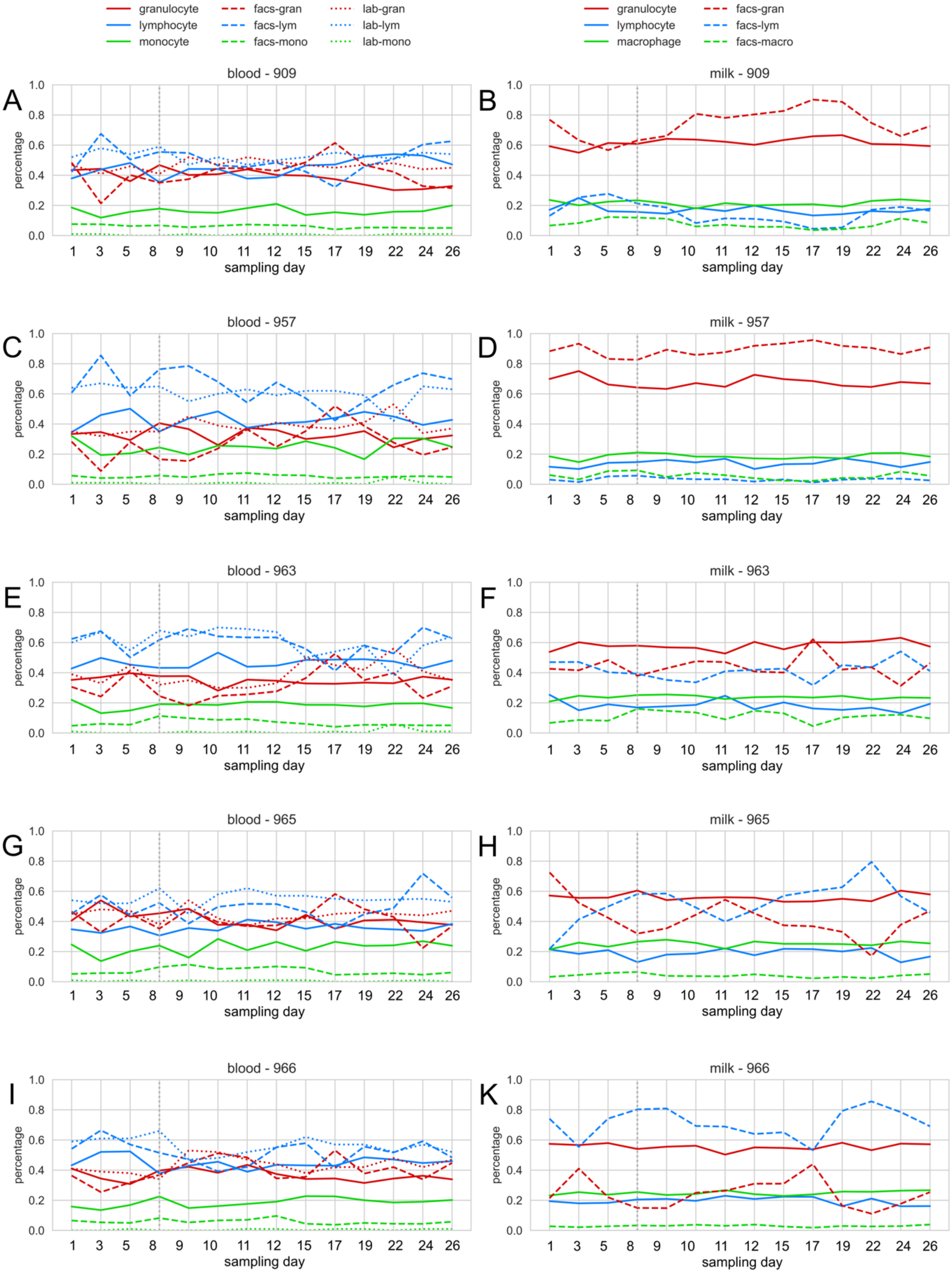

Figure S16. **Cell count progressions over time, DHM results analyzed using Support Vector Machine classification.** A: Blood cells, cow #909. B: Milk cells, cow #909. C: Blood cells, cow #957. D: Milk cells, cow #957. E: Blood cells, cow #963. F: Milk cells, cow #963. G: Blood cells, cow #965. H: Milk cells, cow #965. I: Blood cells, cow #966. K: Milk cells, cow #966.

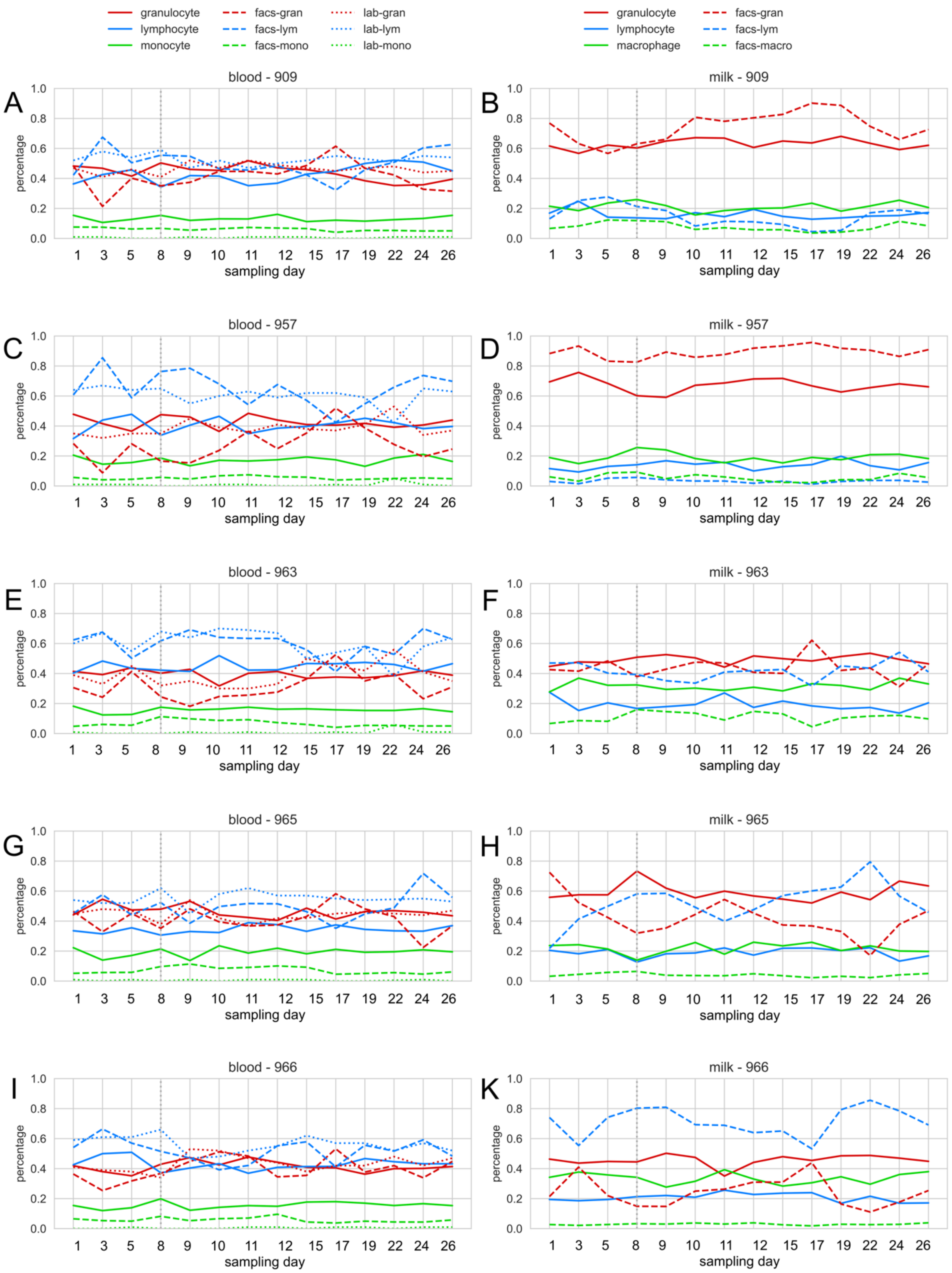

Supplementary Tables

Table S1. **Detailed information about the cows used in this study.** For each sampling day, the days in milk of the cows' current lactation, the milk yield and the SCC values of the morning milking, during which sampling took place, are listed.

Cow	Day	Days in milk	Milk yield of morning milking (kg)	SCC (cells/ml)
#909	1	203	17.69	70200
	3	205	17.22	50240
	5	207	18.74	39360
	8	210	18.95	44320
	9	211	17.96	36720
	10	212	17.44	49520
	11	213	19.02	37040
	12	214	17.45	41520
	15	217	16.04	58720
	17	219	18.85	48160
	19	221	18.61	70080
	22	224	17.71	49200
	24	226	20.22	40000
	26	228	17.42	50000
#957	1	229	13.95	83600
	3	231	13.94	78640
	5	233	12.91	56240
	8	236	13.55	56480
	9	237	13.93	60960
	10	238	13.58	49120
	11	239	13.42	48000
	12	240	12.63	70000
	15	243	12.37	53360
	17	245	13.15	55040
	19	247	12.38	76000
	22	250	13.51	70080
	24	252	12.47	42720

	26	254	13.30	62880
#963	1	197	13.82	91500
	3	199	13.42	47000
	5	201	13.17	52133
	8	204	12.71	108667
	9	205	13.67	67333
	10	206	12.73	60200
	11	207	13.54	54867
	12	208	13.01	52933
	15	211	13.82	30467
	17	213	19.07	60533
	19	215	14.83	50286
	22	218	13.90	84000
	24	220	13.84	66533
	26	222	13.33	62267
#965	1	186	16.67	34400
	3	188	14.13	37900
	5	190	14.81	26550
	8	193	15.25	52500
	9	194	15.08	39300
	10	195	15.90	23200
	11	196	15.87	36350
	12	197	14.49	35500
	15	200	14.69	24850
	17	202	15.18	27050
	19	204	14.77	51942
	22	207	15.61	30514
	24	209	15.85	58500
	26	211	13.77	39350
#966	1	176	14.96	31900
	3	178	15.77	33650
	5	180	16.03	25150

	8	183	16.84	27300
	9	184	14.87	26200
	10	185	16.35	22850
	11	186	16.22	33600
	12	187	14.93	31350
	15	190	13.70	32500
	17	192	15.86	44250
	19	194	15.02	40600
	22	197	15.13	55789
	24	199	15.91	41700
	26	201	15.01	16300

Table S2. **Concentration of viability dye.** Reagent was diluted in DPBS (Dulbecco's Phosphate Buffered Saline, Sigma Aldrich, Co.).

Zombie NIR Fixable Viability Kit (Biolegend, Inc.)	1:800

Table S3. **Concentrations of antibodies.** Reagents were diluted in FACS buffer (DPBS with 2% fetal bovine serum (Sigma Aldrich, Co.) and 0.01 % NaN_3).

Mouse anti-sheep CD45, Biotin, IgG1, clone 1.11.32 (BioRad Laboratories Inc.)	1:250
Mouse anti-bovine CD11b, unlabeled, IgG2b, clone MM10A (Monoclonal Antibody Center, Washington State University, USA)	1:400
Mouse anti-human CD14, BV711, IgG2a, clone M5E2 (Becton, Dickinson and Company)	1:66
Mouse anti-bovine CD4, Alexa Fluor 647, IgG2a, clone CC8 (BioRad Laboratories Inc.)	1:250
Mouse anti-bovine CD8, unlabeled, IgG3, clone CACT130A (Monoclonal Antibody Center, Washington State University, USA)	1:250
Mouse anti-bovine CD21, PE-Cy7, IgG1, clone LT21 (EXBIO Praha, a.s.)	1:200
Mouse anti-bovine CD335, PE, IgG1, clone AKS1 (BioRad Laboratories Inc.)	1:20
Mouse anti-bovine γδ TCR1-N24 δ chain, IgG2b, clone GB21A (Monoclonal Antibody Center, Washington State University, USA)	1:250
Streptavidin BV786 (Becton, Dickinson and Company)	1:800
Rat anti-mouse IgG2b, BV605, clone R12-3 (Becton, Dickinson and Company)	1:1,000
Rat anti-mouse IgG3, BV421, clone R40-82 (Becton, Dickinson and Company)	1:400
Rat anti-mouse IgG2b, FITC, clone m2b-25G4 (Thermo Fisher Scientific Inc.)	1:1,000

Table S4. **Descriptions of features**.

**Feature**	**Description**
Area [µm²]	area of the cell contour
Aspect Ratio [ ]	ratio of the height and width of the minimal fitting rectangle
Biconcavity [ ]	coefficient a1 of fitted discocyte, positive values indicate biconcave, 0 spherical, negative triangular
Circularity [ ]	circularity of the contour by ratio of area and perimeter
Contrast [ ]	intensity contrast between a pixel and its neighbor over the whole image
Correlation [ ]	correlation between a pixel and its neighbor over the whole image
Density [ ]	coefficient a0 of fitted discocyte, measure for the density
Discocyte error [ ]	summed up and normalized difference between cell and fitted discocyte
Dissimilarity [ ]	intensity contrast between a pixel and its neighbor over the whole image
Energy [ ]	sqrt of sum of squared values in glcm
Entropy [ ]	statistical measure of randomness
Homogeneity [ ]	homogeneity of the image i.e. how many dominant gray-tone transitions
Mass Center Shift [µm]	euclidean distance between geometric and mass center
Optical Height Max [rad]	max of the optical height
Optical Height Min [rad]	min of the optical height
Optical Height Mean [rad]	mean of the optical height
Optical Height Std [rad]	standard deviation of the optical height
Radius Max [µm]	max of the radius between contour and geometric center
Radius Min [µm]	min of the radius between contour and geometric center

Radius Mean [µm]	mean of the radius between contour and geometric center
Radius Std [µm]	standard deviation of the radius between contour and geometric center
Solidity [ ]	ratio of contour area to its convex hull area
Steepness [ ]	coefficient a2 of fitted discocyte, positive values indicate steeper edges
Volume [µm³]	optical volume of the cell

Table S5. **Combinations explored for each classifier.**

Random Forest<br>• Number of estimators: 100, 500, 1000<br>• Maximum number of features: 2, 4, 8<br>• Maximum depth: None, 4, 10, 16, 20
Support Vector Machine<br>• C: 0.3, 0.4, 0.5, 0.7, 1.0<br>• Kernel: "sigmoid", "rbf", "linear"<br>• Gamma: 0.004, 0.005, 0.006, 0.010, 0.050, 0.1,00 0.500
k-Nearest Neighbor<br>• Number of neighbors: 1, 3, 5, 10, 30, 50, 100<br>• Weights: "uniform", "distance"<br>• P: 1, 2, 3

Table S6. **Specificity of trained classifiers to identify sorted blood cells of test set.**

**Classifier**	**Set**	**Specificity (granulocytes)**	**Specificity (lymphocytes)**	**Specificity (monocytes)**	**Specificity (all cells)**
kNN	train	1.0	1.0	1.0	1.0
kNN	test	0.8888	0.9619	0.9198	0.9044
RFs	train	1.0	1.0	1.0	1.0
RFs	test	0.9226	0.9644	0.9492	0.9328
SVM	train	0.9845	0.9781	0.9641	0.9806
SVM	test	0.9091	0.9721	0.951	0.9248

Table S7. **Sensitivity of trained classifiers to identify sorted blood cells of test set.**

**Classifier**	**Set**	**Sensitivity (granulocytes)**	**Sensitivity (lymphocytes)**	**Sensitivity (monocytes)**	**Sensitivity (all cells)**
kNN	train	1.0	1.0	1.0	1.0
kNN	test	0.887	0.8588	0.7709	0.866
RFs	train	1.0	1.0	1.0	1.0
RFs	test	0.9164	0.9373	0.8031	0.9033
SVM	train	0.9393	0.9842	0.938	0.9461
SVM	test	0.9262	0.9125	0.8099	0.9074

Table S8. **Precision of trained classifiers to identify sorted blood cells of test set.**

**Classifier**	**Set**	**Precision (granulocytes)**	**Precision (lymphocytes)**	**Precision (monocytes)**	**Precision (all cells)**
kNN	train	1.0	1.0	1.0	1.0
kNN	test	0.9497	0.8034	0.6165	0.8795
RFs	train	1.0	1.0	1.0	1.0
RFs	test	0.9656	0.8268	0.7255	0.9099
SVM	train	0.993	0.8919	0.813	0.9516
SVM	test	0.9602	0.8558	0.7342	0.9118

Table S9. **Specificity of trained classifiers to identify sorted milk cells of test set.**

**Classifier**	**Set**	**Specificity (granulocytes)**	**Specificity (lymphocytes)**	**Specificity (macrophages)**	**Specificity (all cells)**
kNN	train	1.0	1.0	1.0	1.0
kNN	test	0.7981	0.9004	0.885	0.8243
RFs	train	1.0	1.0	1.0	1.0
RFs	test	0.8126	0.9236	0.9176	0.8419
SVM	train	0.9876	0.9713	0.9598	0.9824
SVM	test	0.7487	0.9407	0.9281	0.7992

Table S10. **Sensitivity of trained classifiers to identify sorted milk cells of test set.**

**Classifier**	**Set**	**Sensitivity (granulocytes)**	**Sensitivity (lymphocytes)**	**Sensitivity (macrophages)**	**Sensitivity (all cells)**
kNN	train	1.0	1.0	1.0	1.0
kNN	test	0.8273	0.6692	0.3747	0.7593
RFs	train	1.0	1.0	1.0	1.0
RFs	test	0.8789	0.7459	0.3852	0.8119
SVM	train	0.9386	0.9389	0.911	0.9363
SVM	test	0.8958	0.7245	0.3615	0.8183

Table S11. **Precision of trained classifiers to identify sorted milk cells of test set.**

**Classifier**	**Set**	**Precision (granulocytes)**	**Precision (lymphocytes)**	**Precision (macrophages)**	**Precision (all cells)**
kNN	train	1.0	1.0	1.0	1.0
kNN	test	0.9177	0.5991	0.2363	0.8007
RFs	train	1.0	1.0	1.0	1.0
RFs	test	0.9274	0.6848	0.3074	0.8295
SVM	train	0.9954	0.8753	0.6776	0.9472
SVM	test	0.9066	0.731	0.3231	0.8241

Table S12. **Outcome of classification of blood cells of unknown test subject using k-Nearest Neighbor classification.**

	**Accuracy**	**Specificity**	**Sensitivity**
Cow #909	0.7869	0.8010	0.8226
Cow #963	0.6854	0.79607	0.7609
Cow #965	0.7413	0.8536	0.8451
**Mean**	0.7379	0.8169	0.8096
**SD**	0.0415	0.0260	0.0356

Table S13. **Outcome of classification of milk cells of unknown test subject using k-Nearest Neighbor classification.**

	**Accuracy**	**Specificity**	**Sensitivity**
Cow #909	0.5486	0.7852	0.6583
Cow #963	0.6280	0.7878	0.7842
Cow #965	0.5147	0.7064	0.7334
**Mean**	0.5638	0.7598	0.7253
**SD**	0.0475	0.0378	0.0517

Table S14. **Outcome of classification of blood cells of unknown test subject using Random Forest classification.**

	**Accuracy**	**Specificity**	**Sensitivity**
Cow #909	0.8108	0.7937	0.8613
Cow #963	0.7167	0.8247	0.7823
Cow #965	0.7430	0.8484	0.8731
**Mean**	0.7568	0.8223	0.8389
**SD**	0.0396	0.0224	0.0403

Table S15. **Outcome of classification of milk cells of unknown test subject using Random Forest classification.**

	**Accuracy**	**Specificity**	**Sensitivity**
Cow #909	0.5925	0.7874	0.7173
Cow #963	0.6781	0.8177	0.8288
Cow #965	0.5692	0.7329	0.7846
**Mean**	0.6133	0.7794	0.7769
**SD**	0.0468	0.0351	0.0458

Table S16. **Outcome of classification of blood cells of unknown test subject using Support Vector Machine classification.**

	**Accuracy**	**Specificity**	**Sensitivity**
Cow #909	0.8259	0.8202	0.8732
Cow #963	0.7102	0.8090	0.7918
Cow #965	0.7287	0.8213	0.8649
**Mean**	0.7549	0.8168	0.8433
**SD**	0.0507	0.0056	0.0366

Table S17. **Outcome of classification of milk cells of unknown test subject using Support Vector Machine classification.**

	**Accuracy**	**Specificity**	**Sensitivity**
Cow #909	0.5860	0.7489	0.7314
Cow #963	0.6730	0.8066	0.8317
Cow #965	0.5175	0.6383	0.7987
**Mean**	0.5922	0.7313	0.7873
**SD**	0.0636	0.0698	0.0417

Table S18. **Comparison of DHM results to FACS results for blood cells.**

Classifier	Cow	Cell Type	MAE	RMSE	MRE
kNN	909	granulocyte	0.0789	0.1047	0.2099
kNN	909	lymphocyte	0.0967	0.1223	0.1855
kNN	909	monocyte	0.1213	0.1223	2.0668
kNN	957	granulocyte	0.1143	0.1438	0.6559
kNN	957	lymphocyte	0.2323	0.2603	0.3358
kNN	957	monocyte	0.1702	0.1713	3.2874
kNN	963	granulocyte	0.089	0.1076	0.3356
kNN	963	lymphocyte	0.1638	0.179	0.2619
kNN	963	monocyte	0.1286	0.1301	2.1646
kNN	965	granulocyte	0.0643	0.0972	0.183
kNN	965	lymphocyte	0.1481	0.171	0.2811
kNN	965	monocyte	0.1542	0.1573	2.444
kNN	966	granulocyte	0.0596	0.0742	0.1457
kNN	966	lymphocyte	0.0962	0.1105	0.1765
kNN	966	monocyte	0.1393	0.1409	2.6294
kNN	all	all	0.1238	0.1395	1.0242
kNN	all	granulocyte	0.0812	0.1055	0.306
kNN	all	lymphocyte	0.1474	0.1686	0.2482
kNN	all	monocyte	0.1427	0.1444	2.5184
RFs	909	granulocyte	0.0823	0.1106	0.2209
RFs	909	lymphocyte	0.0945	0.1148	0.1864
RFs	909	monocyte	0.1023	0.1054	1.7466
RFs	957	granulocyte	0.1015	0.1328	0.581
RFs	957	lymphocyte	0.2289	0.2576	0.3309
RFs	957	monocyte	0.1935	0.1979	3.7012
RFs	963	granulocyte	0.0875	0.1062	0.325
RFs	963	lymphocyte	0.1477	0.1638	0.2365
RFs	963	monocyte	0.1179	0.1211	1.9989
RFs	965	granulocyte	0.0692	0.1029	0.1908
RFs	965	lymphocyte	0.1423	0.1681	0.2681
RFs	965	monocyte	0.1565	0.1644	2.5288
RFs	966	granulocyte	0.0629	0.0798	0.1528
RFs	966	lymphocyte	0.0815	0.0944	0.1509
RFs	966	monocyte	0.1271	0.1314	2.4283
RFs	all	all	0.1197	0.1368	1.0031

RFs	all	granulocyte	0.0807	0.1065	0.2941
RFs	all	lymphocyte	0.139	0.1597	0.2346
RFs	all	monocyte	0.1395	0.1441	2.4808
SVM	909	granulocyte	0.0787	0.1056	0.2287
SVM	909	lymphocyte	0.1016	0.1235	0.1967
SVM	909	monocyte	0.0705	0.0727	1.2094
SVM	957	granulocyte	0.17	0.1929	0.9226
SVM	957	lymphocyte	0.249	0.277	0.3612
SVM	957	monocyte	0.118	0.12	2.2777
SVM	963	granulocyte	0.1066	0.128	0.4161
SVM	963	lymphocyte	0.1615	0.1779	0.258
SVM	963	monocyte	0.0908	0.0934	1.5524
SVM	965	granulocyte	0.0826	0.1088	0.2395
SVM	965	lymphocyte	0.1542	0.1774	0.2924
SVM	965	monocyte	0.1216	0.1275	1.9711
SVM	966	granulocyte	0.0575	0.0688	0.1557
SVM	966	lymphocyte	0.0947	0.1076	0.1743
SVM	966	monocyte	0.0976	0.101	1.8683
SVM	all	all	0.117	0.1321	0.8083
SVM	all	granulocyte	0.099	0.1208	0.3925
SVM	all	lymphocyte	0.1522	0.1727	0.2565
SVM	all	monocyte	0.0997	0.1029	1.7758

Table S19. **Comparison of DHM results to FACS results for milk cells.**

Classifier	Cow	Cell Type	MAE	RMSE	MRE
kNN	909	granulocyte	0.2164	0.2304	0.2822
kNN	909	lymphocyte	0.0751	0.0894	0.8746
kNN	909	macrophage	0.1805	0.1816	2.7387
kNN	957	granulocyte	0.3159	0.3178	0.3534
kNN	957	lymphocyte	0.1556	0.1569	5.6614
kNN	957	macrophage	0.1793	0.1801	4.0374
kNN	963	granulocyte	0.0553	0.0709	0.1307
kNN	963	lymphocyte	0.1635	0.1751	0.3802
kNN	963	macrophage	0.1856	0.1882	2.013
kNN	965	granulocyte	0.0953	0.1212	0.2934
kNN	965	lymphocyte	0.2664	0.2876	0.4863
kNN	965	macrophage	0.2354	0.236	6.463
kNN	966	granulocyte	0.1889	0.2103	1.0475
kNN	966	lymphocyte	0.4447	0.4549	0.6164
kNN	966	macrophage	0.2704	0.2706	9.5458
kNN	all	all	0.2019	0.2114	2.3283
kNN	all	granulocyte	0.1743	0.1901	0.4214
kNN	all	lymphocyte	0.2211	0.2328	1.6038
kNN	all	macrophage	0.2102	0.2113	4.9596
RFs	909	granulocyte	0.133	0.1515	0.1698
RFs	909	lymphocyte	0.0566	0.0662	0.5912
RFs	909	macrophage	0.137	0.1389	2.1039
RFs	957	granulocyte	0.2176	0.2205	0.243
RFs	957	lymphocyte	0.1059	0.1079	3.898
RFs	957	macrophage	0.1308	0.1319	2.9748
RFs	963	granulocyte	0.1446	0.1613	0.3592
RFs	963	lymphocyte	0.2351	0.2451	0.5552
RFs	963	macrophage	0.1281	0.131	1.4108
RFs	965	granulocyte	0.1617	0.1846	0.5006
RFs	965	lymphocyte	0.3304	0.3561	0.5987
RFs	965	macrophage	0.2103	0.2111	5.7372
RFs	966	granulocyte	0.3113	0.3266	1.6323
RFs	966	lymphocyte	0.5166	0.5262	0.7185
RFs	966	macrophage	0.2186	0.219	7.7299
RFs	all	all	0.2025	0.2119	1.9482

RFs	all	granulocyte	0.1937	0.2089	0.581
RFs	all	lymphocyte	0.2489	0.2603	1.2723
RFs	all	macrophage	0.1649	0.1664	3.9913
SVM	909	granulocyte	0.1209	0.14	0.154
SVM	909	lymphocyte	0.0571	0.067	0.5638
SVM	909	macrophage	0.1333	0.1357	2.0458
SVM	957	granulocyte	0.2213	0.2261	0.2471
SVM	957	lymphocyte	0.1048	0.1082	3.8892
SVM	957	macrophage	0.1356	0.1383	3.0574
SVM	963	granulocyte	0.0781	0.094	0.1903
SVM	963	lymphocyte	0.2241	0.2356	0.528
SVM	963	macrophage	0.2060	0.211	2.2439
SVM	965	granulocyte	0.1959	0.223	0.5923
SVM	965	lymphocyte	0.3305	0.3554	0.6008
SVM	965	macrophage	0.1781	0.1826	5.0323
SVM	966	granulocyte	0.212	0.2393	1.1901
SVM	966	lymphocyte	0.5054	0.5158	0.7021
SVM	966	macrophage	0.3067	0.3087	10.815
SVM	all	all	0.2007	0.2007	2.1235
SVM	all	granulocyte	0.1656	0.1656	0.4747
SVM	all	lymphocyte	0.2444	0.2444	1.2568
SVM	all	macrophage	0.1919	0.1919	4.639

Table S20. **Comparison of DHM results to results of external laboratory for blood cells.**

**Classifier**	**Cow**	**Cell Type**	**MAE**	**RMSE**	**MRE**
kNN	909	granulocyte	0.0864	0.0951	0.1841
kNN	909	lymphocyte	0.0983	0.1143	0.1835
kNN	909	monocyte	0.1762	0.1767	inf
kNN	957	granulocyte	0.0549	0.0772	0.1329
kNN	957	lymphocyte	0.1903	0.2019	0.3059
kNN	957	monocyte	0.2151	0.216	inf
kNN	963	granulocyte	0.0655	0.0862	0.1537
kNN	963	lymphocyte	0.1663	0.1808	0.2669
kNN	963	monocyte	0.1871	0.1882	inf
kNN	965	granulocyte	0.0445	0.0489	0.0999
kNN	965	lymphocyte	0.1983	0.2039	0.3568
kNN	965	monocyte	0.221	0.2224	inf
kNN	966	granulocyte	0.0698	0.0806	0.1566
kNN	966	lymphocyte	0.1312	0.1454	0.2273
kNN	966	monocyte	0.1924	0.193	inf
kNN	all	all	0.1451	0.1487	0.2068
kNN	all	granulocyte	0.0639	0.0776	0.1454
kNN	all	lymphocyte	0.1569	0.1693	0.2681
kNN	all	monocyte	0.1984	0.1993	
RFs	909	granulocyte	0.0919	0.1	0.1966
RFs	909	lymphocyte	0.0818	0.1008	0.1525
RFs	909	monocyte	0.1571	0.1589	inf
RFs	957	granulocyte	0.0707	0.0967	0.171
RFs	957	lymphocyte	0.1856	0.2001	0.2977
RFs	957	monocyte	0.2384	0.2418	inf
RFs	963	granulocyte	0.0682	0.0924	0.1583
RFs	963	lymphocyte	0.15	0.1651	0.2418
RFs	963	monocyte	0.1763	0.1779	inf
RFs	965	granulocyte	0.055	0.0604	0.1234
RFs	965	lymphocyte	0.1925	0.199	0.3456
RFs	965	monocyte	0.2221	0.2262	inf
RFs	966	granulocyte	0.076	0.0861	0.1712
RFs	966	lymphocyte	0.1124	0.1299	0.1934
RFs	966	monocyte	0.1803	0.1823	inf

RFs	all	all	0.1372	0.1478	0.2052
RFs	all	granulocyte	0.0724	0.0871	0.1641
RFs	all	lymphocyte	0.1445	0.159	0.2462
RFs	all	monocyte	0.1949	0.1974	inf
SVM	909	granulocyte	0.049	0.061	0.1078
SVM	909	lymphocyte	0.1007	0.1165	0.1886
SVM	909	monocyte	0.1253	0.1265	inf
SVM	957	granulocyte	0.0645	0.0799	0.1723
SVM	957	lymphocyte	0.2042	0.2198	0.3263
SVM	957	monocyte	0.1629	0.1646	inf
SVM	963	granulocyte	0.0669	0.0811	0.1709
SVM	963	lymphocyte	0.1649	0.1793	0.2653
SVM	963	monocyte	0.1492	0.1508	inf
SVM	965	granulocyte	0.0334	0.0439	0.0794
SVM	965	lymphocyte	0.2058	0.211	0.3702
SVM	965	monocyte	0.1881	0.1905	inf
SVM	966	granulocyte	0.0396	0.0503	0.091
SVM	966	lymphocyte	0.1308	0.1441	0.2273
SVM	966	monocyte	0.1507	0.1521	inf
SVM	all	all	0.1224	0.1314	0.1999
SVM	all	granulocyte	0.0507	0.0633	0.1243
SVM	all	lymphocyte	0.1613	0.1741	0.2755
SVM	all	monocyte	0.1553	0.1569	inf
